# Respiratory Syncytial Virus in Adult Patients at a Tertiary Care Hospital in Germany: Clinical Features and Molecular Epidemiology of the Fusion Protein in the Severe Respiratory Season of 2022/2023

**DOI:** 10.3390/v16060943

**Published:** 2024-06-12

**Authors:** Mario Hönemann, Melanie Maier, Armin Frille, Stephanie Thiem, Sandra Bergs, Thomas C. Williams, Vicente Mas, Christoph Lübbert, Corinna Pietsch

**Affiliations:** 1 Virology Department, Institute of Medical Microbiology and Virology, Leipzig University Hospital, Johannisalle 30, 04103 Leipzig, Germany; 2 Interdisciplinary Center for Infectious Diseases, Leipzig University Hospital, Liebigstrasse 20, 04103 Leipzig, Germany; 3 Department of Respiratory Medicine, Leipzig University Hospital, Liebigstrasse 20, 04103 Leipzig, Germany; 4 Child Life and Health, University of Edinburgh, Royal Hospital for Children and Young People, 50 Little France Crescent, Edinburgh EH16 4TJ, UK; 5 Centro Nacional de Microbiología and CIBER de Enfermedades Respiratorias, Instituto de Salud Carlos III, C/ Sinesio Delgado, 4, 28029 Madrid, Spain; 6 Division of Infectious Diseases and Tropical Medicine, Department of Medicine I, Leipzig University Hospital, Liebigstrasse 20, 04103 Leipzig, Germany

**Keywords:** RSV, molecular epidemiology, fusion protein, respiratory infections, respiratory viruses

## Abstract

Following an interseasonal rise in mainly pediatric respiratory syncytial virus (RSV) cases in Germany in 2021, an exceptionally high number of adult cases was observed in the subsequent respiratory season of 2022/2023. The aim of this study was to compare the clinical presentation of RSV infections in the pre- and post-SARS-CoV-2 pandemic periods. Additionally, the local epidemiology of the RSV fusion protein was analyzed at a molecular genetic and amino acid level. RSV detections in adults peaked in calendar week 1 of 2023, 8 weeks earlier than the earliest peak observed in the three pre-pandemic seasons. Although the median age of the adult patients was not different (66.5 vs. 65 years), subtle differences between both periods regarding comorbidities and the clinical presentation of RSV cases were noted. High rates of comorbidities prevailed; however, significantly lower numbers of patients with a history of lung transplantation (*p* = 0.009), chronic kidney disease (*p* = 0.013), and immunosuppression (*p* = 0.038) were observed in the 2022/2023 season. In contrast, significantly more lower respiratory tract infections (*p* < 0.001), in particular in the form of pneumonia (*p* = 0.015) and exacerbations of obstructive lung diseases (*p* = 0.008), were detected. An ICU admission was noted for 23.7% of all patients throughout the study period. Sequence analysis of the fusion protein gene revealed a close phylogenetic relatedness, regardless of the season of origin. However, especially for RSV-B, an accumulation of amino acid point substitutions was noted, including in antigenic site Ø. The SARS-CoV-2 pandemic had a tremendous impact on the seasonality of RSV, and the introduction of new vaccination and immunization strategies against RSV warrants further epidemiologic studies of this important pathogen.

## 1. Introduction

Respiratory syncytial virus (RSV) is one of the most widespread respiratory pathogens and affects all age groups across the population [1]. Besides mild upper respiratory tract infections (URTIs), RSV can especially cause severe lower respiratory tract infections (LRTIs) in infants and young children below the age of two years [2]. RSV infections in adults seem to predominantly occur in the form of mild URTIs [3]. However, severe disease courses with subsequent hospitalization and the development of LRTIs, i.e., in the form of pneumonia, are frequently observed especially in high-risk patient groups [4,5,6,7,8,9]. In addition to age, comorbidities may result in increased susceptibility to infections with a higher risk for severe disease progression. These include chronic diseases affecting the lungs (chronic obstructive pulmonary disease [COPD]), cardiovascular system, kidneys, liver, central nervous system (stroke), endocrine system (diabetes mellitus), and obesity, as well as other co-factors, such as immunosuppressive drugs (i.e., in the context of solid organ transplantation) [10,11,12,13].

RSV is a negative-stranded RNA virus belonging to the genus *Orthopneumovirus* in the family *Pneumoviridae*. In Europe, infections typically occur in late fall and the winter months with a peak in February [14]. Based on genetic and antigenic diversity [14,15], RSV can be subdivided into two subtypes, RSV-A and RSV-B. The virion contains a lipid envelope with embedded glycoproteins, most notably the fusion protein (F) and the attachment glycoprotein (G). The G protein initiates cell binding, while the F protein mediates fusion of the virion with host cells.

Recent proposals for revised genotype descriptions identified the G ectodomain as the lowest common denominator suitable for RSV genotyping [16,17,18]. However, only a narrow genotype spectrum was observed in recent epidemiological studies, as a global predominance of single genotypes of RSV-A and -B [4,16,17,18,19] has evolved and was even further accelerated by the contact restriction measures in the wake of the SARS-CoV-2 pandemic [20,21]. At the same time, due to recent approvals (i.e. FDA and EMA) of several RSV vaccines intended for pregnant women and adults older than 60 years of age and the new highly potent monoclonal antibody nirsevimab for the RSV prophylaxis of neonates [22,23,24], in addition to the monoclonal antibody palivizumab, the clinical importance and need for molecular surveillance of the fusion protein has vastly increased since 2023. The structure of the fusion protein is highly complex [25] and features a conformational change between a metastable prefusion conformation [26] when incorporated into virus particles and a stable postfusion conformation [27] after membrane binding and fusion. Antibody binding to the prefusion confirmation may be associated with a highly potent neutralization of RSV [28]. Six antigenic sites were identified on the fusion protein, of which three are present only on the prefusion confirmation (sites Ø (zero), II, and V) and three on both conformational states (sites I, II, and IV). Thus far, particular clinical importance can be ascribed to site II, which represents the binding site of palivizumab [29], and site Ø, the main target region for the recently approved vaccines and nirsevimab [30].

Following behavioral changes and the implementation of extensive non-pharmaceutical interventions (NPIs) [20] in 2020 due to the SARS-CoV-2 pandemic, which included nationwide lockdowns with the subsequent closing of schools, daycare centers, and cultural events, RSV activity was greatly reduced during the winter of 2020/2021. Following the easing of NPIs, an interseasonal rise in RSV cases, especially in children and younger adults, was observed in the summer and fall of 2021 [4], while an upsurge in older adults was observed with a delay of one year in 2022 and 2023.

The aim of this study was to characterize the clinical significance of RSV infections of adult patients between seasons 2017/2018, 2018/2019, and 2019/2020 before the SARS-CoV-2 pandemic and the 2022/2023 season. Additionally, the local molecular F gene epidemiology between 2017 and 2023 was analyzed.

## 2. Materials and Methods

### 2.1. Sample Collection and Clinical Data

From October 2017 to September 2023, 17,251 respiratory samples from 9997 adult (≥18 years) in- and outpatients were collected and tested for viral respiratory infections at the University Hospital of Leipzig, Germany.

Samples included nasal and/or naso-oropharyngeal swabs (44.7%, *n* = 7708), throat rinsing fluids (30.1%, *n* = 5200), bronchoalveolar lavage fluids (15.2%, *n* = 2630), tracheal secretions (8.8%, *n* = 1523), and sputum samples (1.1%, *n* = 190). Testing was initiated at the discretion of the responsible physician. To avoid bias caused by follow-up samples, re-testing within six weeks after initial detection was excluded. For this retrospective observational study, data relating to underlying medical conditions, comorbidities, and clinical parameters from the day of RSV detection were retrieved from patient charts. In the case of missing clinical information, the designation “(*n*/total)” indicates the respective cases for the total amount of available data. A body temperature > 38.0 °C was categorized as fever. The classification of URTIs and LRTIs was carried out according to the International Statistical Classification of Diseases and Related Health Problems (ICD-10-WHO) [31] and the diagnoses and information listed in the patients’ records. Patients with any of the following conditions were considered immunocompromised: receiving active chemotherapy for cancer, chronic neutropenia (<1500/µL for more than three months), receiving steroids or other immunomodulatory medications prior to visit/admission, aplasia of the thymus gland, or reported HIV infection. Furthermore, comorbidities were classified according to the age-adjusted Charlson comorbidity index (CCI) [32]. Asthma, COPD, and Asthma-COPD Overlap Syndrome (ACOS) were summarized as obstructive lung disease (OLD). Anticholinergics and β2-adrenergic agonists (i.e., ipratropium bromide and salbutamol) were classified as bronchodilators. Bacterial and fungal pathogens were cultivated with standard microbiological techniques and considered co-infections if detection from respiratory samples or blood was documented. Viral co-infections were assessed through a multiplex test for respiratory viruses (see below) and established routine laboratory protocols for CMV [33,34], HSV [35], and VZV [36]. SARS-CoV-2 was analyzed with a commercially available SARS-CoV-2 RT-PCR assay for the Alinity m analyzer (Abbott, Chicago, IL, USA) targeting the viral RdRp- and N-genes. 

### 2.2. Saisonality, Detection Intervals, Peak Detection

A respiratory season was defined as starting on October 1st and ending on September 30th of the following year. Deviating from this definition, RSV cases between August 2021 and February 2022 were considered to belong to the 2021/2022 season. Additionally, cases from September 2022 were considered as belonging to the 2022/2023 season. For the sake of this study, the main detection interval, equaling the epidemic period, was defined as the period between the first of two subsequent weeks and the last of two subsequent weeks where at least two RSV cases were identified per week. The peak detection of RSV is equivalent to the week/weeks where the most RSV cases were identified.

### 2.3. Meteorological Data

The data for the mean monthly air temperatures (Leipzig/Halle; Station ID: 2932) were obtained from the Climate Data Centre (CDC) of the Deutscher Wetterdienst (DWD) [37].

### 2.4. Nucleic Acid (NA) Extraction and RSV Detection

Total NA was extracted from 200 μL of the respiratory samples using the DNA and Viral NA Small Volume Kit on a MagNA Pure 96 instrument (both Roche, Mannheim, Germany) according to the manufacturer’s instructions. NAs were stored in aliquots at −80 °C until further use. The presence of genomes of common respiratory viruses, including influenza viruses A and B, RSV-A and -B, parainfluenza viruses 1 to 4, human coronaviruses (including 229E, NL63, OC43, and HKU1), human metapneumoviruses, adenoviruses, human bocaviruses, rhinoviruses, and enteroviruses, was assessed using a multiplex panel assay (NxTAG RPP, Luminex corporation, Austin, TX, USA) according to the manufacturer’s instructions. Samples that reacted to either one or both RSV targets of the assay were further analyzed.

### 2.5. Phylogenetic Analysis of the RSV F Gene Sequence

The complete viral fusion protein gene (F gene) was amplified using a protocol optimized for contemporary RSV genotypes. The resulting amplicons spanned a fragment of approximately 2150 bp for both RSV-A and RSV-B after the final nested PCR step. Sequences were generated using either of the following two approaches. (1) Sanger Sequencing was performed using the BigDye Terminator Sequencing Kit v1.1 and an ABI 3500 Genetic Analyzer (both Applied Biosystems, Foster City, CA, USA). (2) Oxford Nanopore Sequencing was performed using the Rapid Barcoding Kit 96, R9.4.1 flow cells and a GridION Mk1 (all Oxford Nanopore Technologies Ltd., Oxford, UK). Reads were basecalled in real time with high accuracy and re-basecalled with super accuracy. Reads with a length above 600 nt were mapped to reference sequences KY654514 (RSV-A) and KY684758 (RSV-B). For a detailed description of the reaction conditions and primers, see Supplementary Tables S1–S7. The sequencing approach was applied to at least ten randomly selected samples of the four seasons before 2022/2023 that were already genotyped in the G gene [4]. For the 2022/2023 season, the sequencing approach was applied to all RSV-A cases and to randomly selected RSV-B cases, including all fatal cases. The sequences obtained were generated using Geneious Prime software, version 2023.2.1, and submitted to GenBank (accession numbers PP084746-PP084890 and PP354071-PP354073). Separate phylogenetic trees for RSV-A and RSV-B were constructed at the nucleotide level using MEGA software, version 7, based on the maximum likelihood method. Bootstrap analyses were performed with 1000 replicates [38]. For the analysis of the relatedness of clinical isolates of the current study, two phylogenetic trees for RSV-A and RSV-B were constructed, including full-length fusion protein gene consensus sequences derived from reference strains proposed by Goya et al. [16] for the genotype analysis of RSV (Supplementary Tables S8 and S9).

### 2.6. Amino Acid Analysis

The obtained fusion (F) protein sequences were translated in order to compare the analyzed strains on an amino acid (AA) level and to the translated consensus reference strains of genotypes GA2.3.5 and GB5.0.5a. The AA changes were mapped to fusion protein subunits, regions, and antigenic sites according to Mas et al. [25]. The AA residues considered for the antigenic sites are provided in Supplementary Table S10.

### 2.7. Statistical Analysis

Statistical analyses were performed using IBM SPSS Statistics for Windows, version 29.0 (IBM Corp., Armonk, NY, USA). Continuous values were expressed as medians (interquartile range (IQR)) and categorical data as frequencies (percentages). A Mann–Whitney U test was performed to compare continuous variables. A chi-square test or Fisher’s exact test was performed for categorical variables. Brackets indicate parameters that were analyzed in the same contingency table. All tests were two tailed. A *p*-level of <0.05 was considered significant. For post hoc pairwise comparisons of column proportions, a Bonferroni correction for multiple comparisons was applied.

## 3. Results

### 3.1. RSV Detection and Seasonality

**Figure 1:**
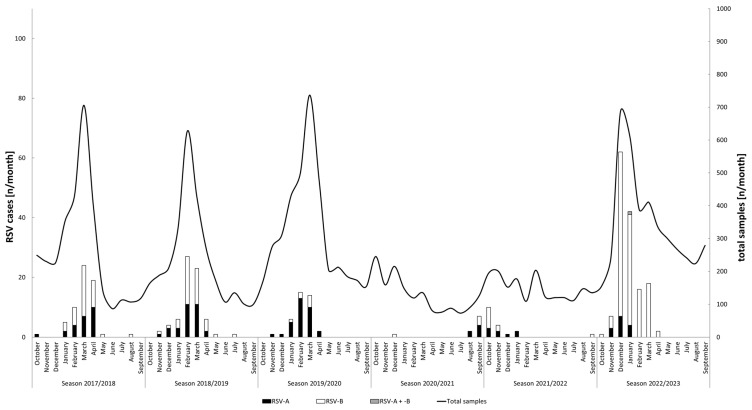
Monthly distribution of total number of samples tested and new RSV cases (*n* = 343) stratified by RSV-A and -B. Note the two different y-axes: the left y-axis refers to the bar charts and shows the absolute number of detected RSV cases while the right y-axis refers to the absolute number of samples tested, as represented by the line graph. The x-axis is labeled according to the definition that was used to define the start and end of a respiratory season.

In total, the presence of RSV RNA was confirmed by RT-PCR in 477 samples originating from 343 unique cases. The absolute numbers of RSV cases detected between 2017 and 2023 stratified by subtype are shown in Figure 1. The main detection intervals for the seasons before 2022/2023 were as follows: week 5 to week 18 of 2018 (season 2017/2018), week 5 to week 17 of 2019 (season 2018/2019), week 4 to week 14 of 2020 (season 2019/2020), and week 37 to week 46 of 2021 (season 2021/2022). For these seasons, weeks with the highest incidence of new RSV cases were weeks 11 and 14 (seven cases each), week 9 (nine cases), weeks 9 and 11 (five cases each), and week 40 (six cases), respectively. For the 2022/2023 season, the main detection interval was observed between week 47 of 2022 (November) and week 13 of 2023 (March). The highest incidence of new cases within a single week was observed in week 1 of 2023 (18 cases). The 2020/2021 season was considered absent.

**Figure 2:**
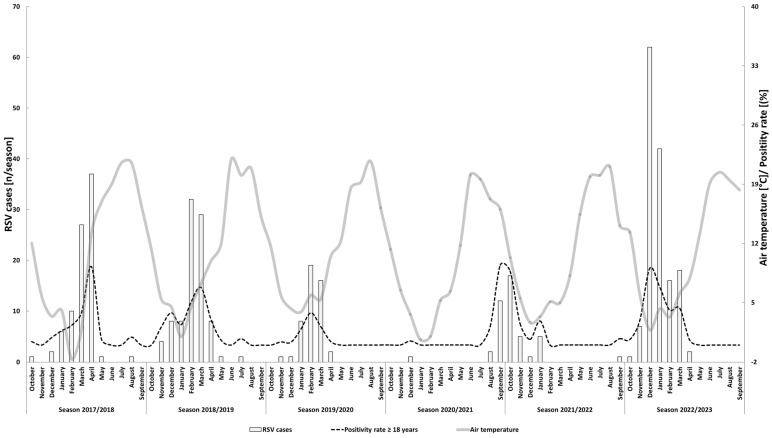
Monthly distribution of RSV cases (*n* = 343), positivity rates of the RSV assay, and mean air temperature during the study period. Note the two different y-axes: the left y-axis refers to the absolute numbers of detected new RSV cases (light gray bars) while the right y-axis refers to the mean temperature of the respective month [°C] (dark gray line) and the positivity rates for adult patients [%] (black dashed line). The x-axis is labeled according to the definition that was used to define the start and end of a respiratory season.

The peak RSV detections coincided with the lowest temperatures of the year (Figure 2), except for the 2021/2022 season. The peak positivity rates for seasons 2017/2018, 2018/2019, 2019/2020, 2021/2022, and 2022/2023 were 9.3%, 6.8%, 3.8%, 9.4%, and 9.1%, respectively.

Two shifts between dominant RSV subtypes were observed during the study period (*p* < 0.001, Table 1). While most cases originated from infections with RSV-A in seasons 2019/2020 and 2021/2022, RSV-B infections were predominantly observed before and thereafter. The patients over the age of 60 predominated in the study population, except for the 2021/2022 season (Table 1).

**Figure 3:**
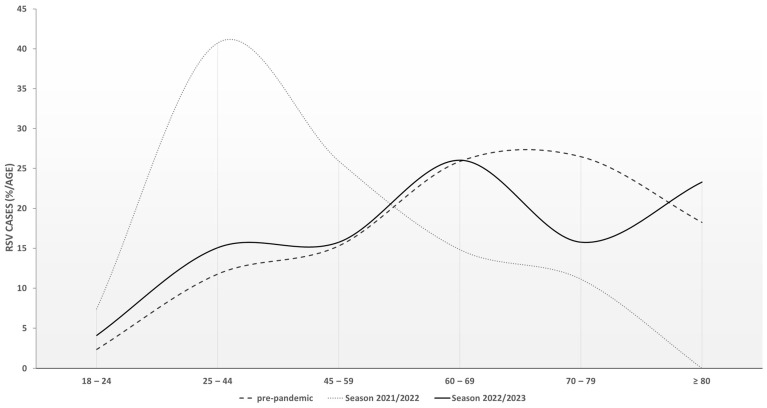
Relative distribution of RSV cases (*n* = 343) stratified by age. The lines represent the relative number of cases detected in the age group indicated. The 2017/2018, 2018/2019, and 2019/2020 seasons were designated “pre-pandemic”.

A relative age stratification into six age groups within RSV cases comparing the pre-pandemic seasons, the 2021/2022 season, and the 2022/2023 season is shown in Figure 3. A significant age difference was detected between those time periods (*p* < 0.001), originating from the high percentage of patients in the 25–44 years group in the 2021/2022 season in comparison to the pre-pandemic cohort (*p* < 0.001) and the patients of the 2022/2023 season (*p* = 0.005).

### 3.2. Study Population and Clinical Features

RSV-related patient characteristics and clinical parameters are presented in Table 2. A comparison between the pre-pandemic seasons (2017/2018, 2018/2019, and 2019/2020) and the 2022/2023 season was performed. Neither the median age nor the sex differed between these periods. The age-adjusted CCI of patients who contracted RSV was not statistically different between the two groups. However, a lower prevalence of patients with chronic kidney failure (26.4 vs. 39.9%, *p* = 0.013) and lung transplantation (0 vs. 4.7%, *p* = 0.009) and patients receiving immunosuppression (25.4 vs. 36.3, *p* = 0.038) were observed in the 2022/2023 season compared with RSV patients in the pre-pandemic seasons. Additionally, for the 2022/2023 season, fever (*p* = 0.032), LRTIs (*p* < 0.001), pneumonia (*p* = 0.015), exacerbation of OLD (*p* = 0.008), and administration of bronchodilators occurred more frequently compared to the pre-pandemic seasons. The detected co-pathogens are listed in Supplementary Table S11. A comparison between the 2021/2022 and 2022/2023 seasons is presented in Supplementary Table S12.

### 3.3. Phylogenetic Analysis

For RSV-A, the F gene amplification approach was performed for 12, 12, 12, 11, and nine cases for the seasons from 2017/2018 to 2022/2023, respectively. For RSV-B, the gene amplification approach was performed for 11, 10, 10, 12, and 48 cases for the seasons from 2017/2018 to 2022/2023, respectively. Within the genotyped subset, respiratory specimens included 111 nasal and/or oropharyngeal swabs (75.5%), 20 throat rinsing fluids (13.6%), 14 bronchoalveolar lavage fluids (9.5%), and two tracheal secretions (1.4%). For all isolates, the coding sequence (CDS) had a length of 1,725 bases, translating into 574 amino acids.

**Figure 4:**
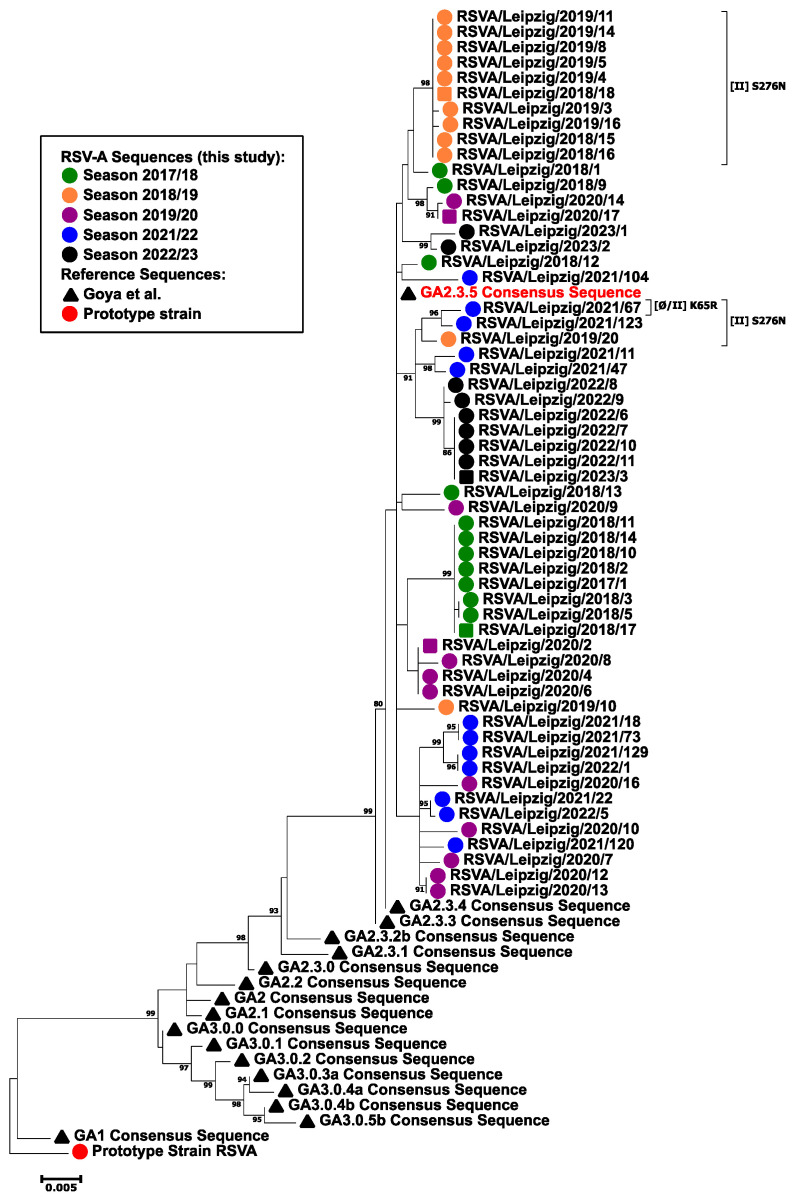
Molecular phylogenetic analysis of the RSV-A F gene by the maximum likelihood method. The evolutionary history was inferred using the maximum likelihood method based on the Tamura–Nei model [39]. The tree with the highest log likelihood (−4744.71) is shown. The percentage of trees in which the associated taxa clustered together is shown next to the branches. Initial tree(s) for the heuristic search were obtained automatically by applying Neighbor-Joining and BioNJ algorithms to a matrix of pairwise distances estimated using the Maximum Composite Likelihood (MCL) approach and then selecting the topology with superior log likelihood value. The tree is drawn to scale, with branch lengths measured in the number of substitutions per site. The analysis involved 75 nucleotide sequences. There were a total of 1725 positions in the final dataset. Evolutionary analyses were conducted in MEGA7 [38]. Only nodes with statistical support > 80% are shown. The following symbols indicate the sequence origin or the season of the indicated strain: dots/squares. Red, RSV-A prototype strain; green: season 2017/2018 isolates; orange, season 2018/2019 isolates; purple, season 2019/2020 isolates; blue, season 2021/2022 isolates; black, season 2022/2023 isolates; squares, fatal cases; black triangle: consensus reference sequences according to Goya et al. [16]. Amino acid changes in sites Ø and II with regard to the GA2.3.5 consensus sequence (red) are given in brackets.

The phylogenetic analysis of the complete F gene revealed a close relationship between all RSV-A isolates analyzed (Figure 4). The pairwise nucleotide identity was 99.5% for the isolates of the 2022/2023 season and 99% for all analyzed sequences. No distinct well-supported cluster was observed for any of the five seasons, as most groupings of different isolates are located in terminal nodes with low statistical support of ancestral nodes. The isolates clustered together with the consensus sequence of GA2.3.5 (Goya et al. [16]), which was also confirmed by a separate phylogenetic analysis carried out with proposed reference strains (Supplementary Figure S1). No distinct cluster of fatal cases was observed.

**Figure 5:**
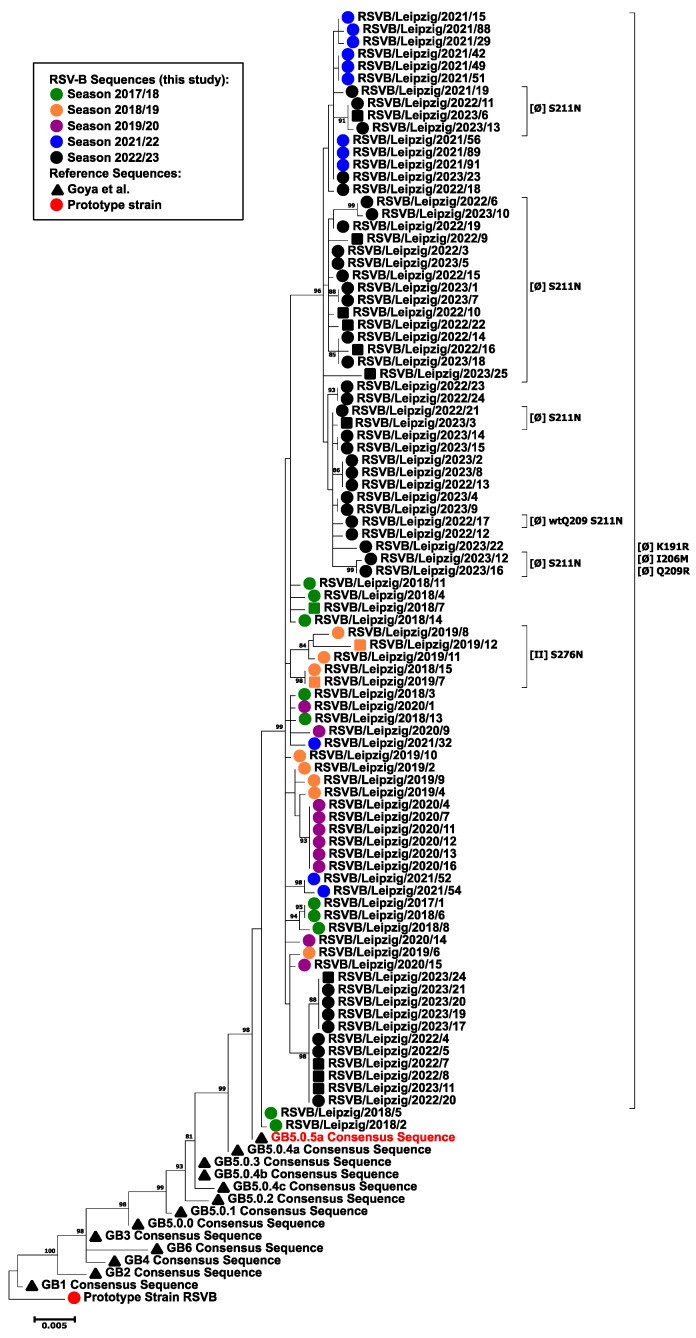
Molecular phylogenetic analysis of the RSV-B F gene by the maximum likelihood method. The evolutionary history was inferred using the maximum likelihood method based on the Tamura–Nei model [39]. The tree with the highest log likelihood (−4220.58) is shown. The percentage of trees in which the associated taxa clustered together is shown next to the branches. Initial tree(s) for the heuristic search were obtained automatically by applying Neighbor-Joining and BioNJ algorithms to a matrix of pairwise distances estimated using the Maximum Composite Likelihood (MCL) approach and then selecting the topology with superior log likelihood value. The tree is drawn to scale, with branch lengths measured in the number of substitutions per site. The analysis involved 105 nucleotide sequences. There were a total of 1725 positions in the final dataset. Evolutionary analyses were conducted in MEGA7 [38]. Only nodes with statistical support > 80% are shown. The following symbols indicate the sequence origin or the season of the indicated strain: dots/squares. Red, RSV-B prototype strain; green: season 2017/2018 isolates; orange, season 2018/2019 isolates; purple, season 2019/2020 isolates; blue, season 2021/2022 isolates; black, season 2022/2023 isolates; squares, fatal cases; black triangle: consensus reference sequences according to Goya et al. [16]. Amino acid changes in sites Ø and II with regard to the GA2.3.5 consensus sequence (red) are given in brackets. Note that for the RSVB/Leipzig/2022/17 strain, no amino acid change was noted at position 209 (wtQ209).

The phylogenetic analysis of the complete F gene revealed a close relationship between all analyzed RSV-B isolates (Figure 5). The pairwise nucleotide identity was 99.4% for the isolates of the 2021/2022 season and 99.3% for all analyzed sequences. The isolates clustered close to the consensus sequence of GB5.0.5a (Goya et al. [16]), which was also confirmed in the separate phylogenetic analysis with proposed reference strains (Supplementary Figure S2). Except for two strains in the 2017/2018 season, the majority of the isolates formed a distinct phylogenetic cluster that corresponded to distinct AA changes with regard to the GA2.3.5 consensus translation (see below). No distinct cluster of fatal cases was observed.

### 3.4. Amino Acid Analysis

The detected AA changes within the fusion protein are depicted in Table 3 and Table 4 (F0 numbering). In comparison to the GA2.3.5 consensus translation, which was used as a reference, no distinct AA exchange was noted that was present in all isolates of the current study. The following AA changes were found in more than five percent of the strains: S276N (22.8%), T12I (22.1%), L20F (15.8%), S105N (14%), A10V (8.8%), and S377N (7%). The majority of AA changes were noted in the F1 subunit (56.7%), especially the signal peptide (41.8%). One AA exchange could be mapped to site Ø, K65R (1.8%), while site II was affected with both K65R and S276N substitutions. In comparison to the GB5.0.5a consensus translation, which was used as a reference, nearly all strains showed three distinct AA changes at positions 191, 206, and 209. In detail, the following substitutions were found in more than five percent of the strains: K191R, I206M (both 97.8%), Q209R (96.7%), S190N (51.7%), S211N, S389P (both 50.6%), K123R (12.1%), and S276N (5.5%). The majority of AA changes were noted in the F2 subunit (93.4%). Five AA changes (I64V, I206M, Q209R, S211N, and E295D) could be mapped to site Ø, affecting 97.8% of all strains, while substitutions in site II were observed in 6.6% of all strains (L273I and S276N). AA changes S190N, S211N, and S389P were highly frequent and only observed in strains from the 2021/22 season onwards.

## 4. Discussion

Following the introduction of NPIs [20,40] and changes in social practices, such as mask wearing in public and increased work from home [39], introduced worldwide and in Germany [41,42,43,44] in the wake of the SARS-CoV-2 pandemic in 2020, the seasonal circulation of other respiratory pathogens, such as RSV, was altered tremendously. Nationwide lockdowns with the subsequent closing of schools, daycare centers, and cultural events led to an exceptional reduction in RSV activity in the winter of 2022/2021. NPIs were eased starting in Germany in May 2021 [45] and tied to the incidence of SARS-CoV-2 infections. However, a more progressive easing of regulations for children and a higher and prolonged adhesion to NPIs in the adult population may represent key contributors to the disparity in the resurgence of RSV infections. While the pediatric population was greatly affected in the 2021/2022 season [4], the majority of the adult population was first affected in the 2022/2023 season.

The start and end points of the pre-pandemic seasons are in line with studies that, for continental Europe, typically define a season with a start in December and an ending in April of the following year [39,40,41]. For Germany, in particular, a season is defined based on the positivity rate (PR) exceeding 5% of RSV real-time RT-PCR detection assays in children between 0 and 4 years of age, as assessed by the national outpatient sentinel surveillance [42] of the Robert Koch Institute (RKI). This age subgroup was chosen because of its high disease burden and clinical importance in order to more accurately correspond to the endemic circulation. According to this definition, the pre-pandemic seasons spanned between weeks 50 to 51 (December) and weeks 11 to 13 (March). The seasonal peaks were reported between weeks four and six (January/February). For the sake of this study, the main detection interval was defined to be framed by each two subsequent weeks with at least two RSV cases per week in order to compensate for a lower catchment population compared to a sentinel surveillance and to additionally compensate for singular cases that would otherwise inflate the analyzed interval. For different study designs and locations, deviating parameters may be needed to generate a robust and comparable detection interval.

This study presents analyses of adult patients that indicate a slightly delayed main detection interval between 2017 and 2020 starting at weeks 4 and 5 (January/February) and ending at weeks 14 to 18 (April/May), which was also confirmed by the seasonal reports of the RKI [46]. The same applies to the peak detections, which were reported between weeks three and six (January/February) for the sentinel cohort and were observed between weeks nine and 14 (February to April) in the adult cohort studied. This distinctive circulation characteristic may be attributed to two main contributing factors. First, the epidemic wave of infection may originate from the infant population and subsequently reach older populations, similar to what was reported for influenza virus infections [47]. Secondly, a delay between the peaks of RSV infections in the general population and hospitalizations may be considered.

The regular RSV circulation pattern observed in the pre-pandemic seasons was remarkably altered with the start of NPIs due to the then-emerging SARS-CoV-2 pandemic. After an abrupt end of the RSV circulation after March 2020, only a few cases were noted for the projected time span of the 2020/2021 season, corresponding to an absent respiratory season [4,46]. After a gradual alleviation of the introduced NPIs, the re-emergence of RSV occurred as a premature season of 2021/2022 and was mainly driven by infections in the pediatric population. According to the definition that is used in Germany, the endemic circulation of RSV started in week 35 (August) and ended in week 50 (December) of 2021, peaking 19 weeks before the usual average (week eight) in week 41 (October). The main detection interval of the studied adult population was observed between weeks 37 (September) and 46 (November) with a peak in week 40 (October), devoid of the temporal delay described above. Furthermore, RSV circulation preceded the lowest air temperatures of the winter of 2021/2022, underlining the unusual properties of this respiratory season.

When compared with the pre-pandemic seasons, a premature start and end were also noted for the 2022/2023 season; however, a shift towards a regular cycle was observed. According to the RKI, in Germany, the endemic circulation occurred between week 41 in 2022 (October) and week 3 in 2023 (January), peaking 13 weeks before the usual average in week 47 (November) [46]. A re-establishment of the delay in RSV detections in the adult cohort was confirmed with a main detection interval between week 47 (November) and week 13 (March). The peak of RSV infections was observed in week 1 (January), eight weeks before the earliest observed peak of one of the pre-pandemic seasons, namely, seasons 2018/2019 and 2019/2020.

The considerably increased amount of RSV cases and altered circulation may highlight the underestimated importance of pre-existing or waning immunity, even in the adult population, as the re-emergence of RSV in 2021 apparently was not caused by major antigenic shifts circumventing a pre-existing immune response [4]. More likely, it seems that an immunologic gap caused by the lack of RSV circulation during the period of intensified contact restrictions was created within the population [48]. Furthermore, the importance of waning immunity and its impact on circulation patterns was previously described for RSV [49] and other respiratory viruses, like enteroviruses [50,51] and influenza B virus [52]. In addition to the apparent differences in the observed amount of RSV cases after the year 2020, age was a major epidemiologic characteristic that differed within the study period. It has to be noted, that the hospitalization rate due to RSV was previously reported to be less variable than e.g. rates observed for influenza over successive seasons. Additionally, the hospitalization burden showed a peak for adults between the ages 65 and 74 [5]. However, while the median age of the study population was 66.5 years in the pre-pandemic seasons, the age group of 25–44-year-old adults was over-represented in the 2021/2021 season, resulting in a significantly lower median age of 49.5 [4], which is in line with observed perturbations in the age composition of adults reported in other studies [53,54,55]. In the 2022/2023 season, the median age of the study population converted back to the pre-pandemic average (65 years). Therefore, excluding 2021/2022, between 65 and 80% of the adults of a respective season were 60 years of age or older and belonged to the target population of the newly introduced RSV vaccines (see below) [23,56]. The reasons for this remarkable disparity in the epidemiological characteristics are likely to be multifactorial and also have implications for the observed differences in the clinical parameters between the pre-pandemic seasons and the 2022/2023 season.

Overall, the prevalence of comorbidities was very high in the studied adult cohort, underlining observations of increased susceptibility to severe disease outcomes subsequent to RSV infections and the hospitalization of high-risk populations [3,5,10,57]. Comorbidities that for RSV have previously been reported to be associated with an increased incidence rate among hospitalized patients or with severe disease progression are, i.e., COPD, asthma, coronary artery disease, congestive heart failure [58,59,60,61], hematologic malignancies and previous stem cell transplantations [6,7], and lung transplantations [8]. Reported incidences of comorbidities were as high as 87% of the study population [62], and mortality after an RSV LRTI was reported to be up to 50% for specific subgroups, i.e., patients after hematopoietic cell transplantation with severe immunodeficiency [7]. 

As the number of comorbidities may vary substantially between different study sites, even within the same study [32], an observed baseline level and composition of comorbidities may be highly specific for the respective region and patient collective that is treated in or connected to each study site, and thus a comparison with other studies at the same site seems warranted. A comparable high number of comorbidities was also observed for infections with other respiratory pathogens at the same hospital, namely, rhinovirus [34], parainfluenza virus type 3 [63], and influenza B virus [52]. The particularly high number of ICU admissions (roughly 25%) of the RSV cases studied was comparable to what was observed for influenza B virus (20–25.8%) [52] and highlights the susceptibility of this patient cohort of developing a severe RSV infection. A comparison between the clinical characteristics of the pre-pandemic seasons and the 2022/2023 season may allow further conclusions about the patient collective at each point in time. Additionally, it may indicate behavioral changes evoked through the SARS-CoV-2 pandemic, i.e., a more liberal utilization of respiratory pathogen tests, including multiplex tests, because of an increased need for pathogen identification and higher awareness of severe viral infections.

After the re-emergence of RSV in 2021/2022, only a small number of adult patients tested positive for RSV at the study site. This may be attributed to an overall lower amount of RSV infections but also to the adherence of severely ill patients to the hospital, as indicated by the high number of immunosuppressed individuals [4]. Unfortunately, the low number of RSV cases in 2021/2022 was also the main obstacle to a robust comparison of clinical data with the two other periods of the current study. In the 2022/2023 season, the study population resembled the pre-pandemic baseline. However, subtle differences were noted. Firstly, the lower prevalence of RSV patients with a history of lung transplantation and immunosuppression may be the result of more stringent adherence to NPIs in specific and at-risk subgroups of adult patients [64]. The same may apply to patients with different stages of chronic kidney injury. Another reason may be indicated by the higher incidences of clinical features like LRTI, pneumonia, exacerbation of OLD, and administration of bronchodilators. As the incidence waves for RSV, influenza, and SARS-CoV-2 infections vastly overlapped in the 2022/2023 season [65], high pressure on the healthcare system was exerted, which may have resulted in a competition for resources and a selection for a higher disease severity in the hospitalized population. However, a waning immunity may also be considered to have an influence on the observed differences in clinical disease severity (see above).

The year 2023 may mark the beginning of a new era of dealing with RSV infections as new highly potent tools become implemented [66] in response to unmet needs for the prophylaxis of otherwise healthy newborns and adults. The vaccine efficacies for adults ≥ 60 years of age in the first RSV season were thereby reported to be up to 85.7% and 94.1%, depending on specific subgroups, for the bivalent [23] and monovalent [56] compositions, respectively. The protection rates for newborns for medically attended severe LRTIs following a maternal immunization were reported to be 81.8% and 69.4%, 90 and 180 days after birth, respectively [22]. For nirsevimab, the efficacy to prevent medically attended RSV-associated LRTI is reported to be 74.5% [22]. Additionally, the SARS-CoV-2 pandemic marked an evolutionary bottleneck, accelerating the notion of a very narrow spectrum of circulating genotypes and lineages [4,16,21], namely, the very closely related lineages GA2.3.5 and GA2.3.6a for RSV-A and GB5.0.5a for RSV-B. Thus, the timing for the introduction of new vaccines and long-acting monoclonal antibodies appears to be optimal.

At the same time, the implementation of new immunization strategies mainly targeting one antigenic site in a broad age range of the population may result in a selection of RSV strains with escape mutations in the fusion protein. In in vitro studies, prolonged selective pressure due to co-cultivation resulted in the selection of escape mutants with decreased binding affinities for various monoclonal antibodies (mABs) [67,68,69,70,71], including palivizumab [72,73]. Furthermore, resistant variants were detected in up to 8.7% of palivizumab recipients [74,75]. Thereby, resistance may not only be conferred by mutations at the binding site but also through distant mutations, resulting in an altered protein conformation [76]. Although generally considered a very conserved protein, the RSV-B isolates showed a greater diversity and drift in the fusion protein than the RSV-A isolates, confirming previous observations of accented differences in antigenic sites of the pre-fusion F [77]. Especially AAs that previously have been ascribed to site Ø [25] were affected in most RSV-B strains, and AA changes accumulated over the study period. In contrast, site II was affected to a much lower extent, and mutations rather appeared in clusters. A continued antigenic drift may have future implications for the efficacy of mABs and vaccines, especially in seasons with a predominance of RSV-B. Thus, molecular and antigenic surveillance of vaccine and mAB non-responders is highly warranted in the future. Furthermore, alternative vaccine strategies [78] and mABs [79] targeting different antigenic sites are needed in order to broaden the immunologic repertoire of the population.

Several limitations of this study should be noted. Notably, the genotyping approach was not applied to all RSV cases and may be a source of selection bias, which might include the circulation of other less common genotypes, especially in the seasons before the SARS-CoV-2 pandemic. However, the authors are confident that the analyzed RSV isolates constitute an adequate representation of the local F gene diversity in the study period and that the protocol applied was usable for contemporary analysis of the F gene, as a narrow genotype spectrum was demonstrated before [4]. Due to the retrospective study design, only associations could be shown, without proof of causality. Patient selection favoring severe cases may have occurred due to the sampling at a tertiary care hospital, which is underlined by the high numbers of pneumonia cases and cases that needed an ICU admission. In addition, the age spectrum of the analyzed patients is likely to underrepresent patients who do not belong to the high-risk groups for severe disease courses and hospitalization. Detection of a further pathogen was considered a co-infection; however, for bacterial pathogens, colonization cannot be ruled out.

## 5. Conclusions

This study reports the epidemiology and associated clinical spectrum of adult RSV cases that were treated at a tertiary care university hospital in Germany between 2017 and 2023. The findings are consistent with other studies and indicate a profound impact of non-pharmaceutical interventions implemented in the wake of the SARS-CoV-2 pandemic. A close phylogenetic relatedness of circulating strains is evidenced by analysis of the F gene. However, an accumulation of amino acid changes was observed, especially for RSV-B strains, and also affected immunologically important antigenic sites. The ongoing implementation of a recently approved monoclonal antibody with an extended half-life [24,30] and vaccination strategies for pregnant women and older adults [22,23,56] in clinical treatment guidelines necessitate a genetic and antigenic analysis of the fusion protein going forward. Additional epidemiologic studies or population-based surveillance programs are warranted, as retained behavioral changes are likely to have an influence on the re-establishment of a regular circulation of respiratory pathogens.

## Figures and Tables

**Table 1:** Sex, age, and RSV species distribution during the study period.

Seasons		2017/2018	2018/2019	2019/2020	2021/2022	2022/2023	Total	*p*-Value
**Age group**								
<60	[% (*n*/total)]	34.4 (21/61)	21.4 (15/70)	35.9 (14/39)	73.1 (19/26)	34.9 (51/146)	35.3 (121/343)	**<0.001**
≥60	[% (*n*/total)]	65. 6 (40/61)	78.6 (55/70)	64.1 (25/39)	26.9 (7/26)	65.1 (95/146)	64.7 (222/343)	
**Sex**								
male	[% (*n*/total)]	67.2 (41/61)	48.6 (34/70)	56.4 (22/39)	57.7 (15/26)	52.1 (76/146)	55.1 (189/343)	n.s.
female	[% (*n*/total)]	32.8 (20/61)	51.4 (36/70)	43.6 (17/39)	42.3 (11/26)	47.9 (70/146)	44.9 (154/343)	
**RSV species**								
RSV-A	[% (*n*/total)]	39.3 (24/61)	44.3 (31/70)	82.1 (32/39)	53.8 (14/26)	8.2 (12/146)	32.9 (113/343)	**<0.001**
RSV-B	[% (*n*/total)]	60.7 (37/61)	55.7 (39/70)	17.9 (7/39)	46.2 (12/26)	91.1 (133/146)	66.8 (229/343)	
mixed	[% (*n*/total)]	-	-	-	-	0.7 (1/146)	0.3 (1/343)	

Analyzed categories are displayed on the column to the left and given as relative and absolute frequencies [% (*n*/total)]. (*n*/total) indicates the respective cases of the total number of cases in the respective season. The *p*-values of the chi-square tests for the contingency tables, including all seasons and subgroups, are indicated. Significant *p*-values for the post hoc pairwise analysis of specific categories (age group) are given in the corresponding paragraph. n.s., not significant.

**Table 2:** Study population and clinical features of the 2022/2023 season and pre-pandemic RSV cases.

		Pre-Pandemic	2022/2023	Total	*p*-Value
**Study population**					
Female	[% (*n*/total)]	42.9 (73/170)	47.9 (70/146)	45.3 (143/316)	n.s.
Male	[% (*n*/total)]	57.1 (97/170)	52.1 (76/146)	54.7 (173/316)	
Age [years]	[median (IQR)] SD]	66.5 (56–77)	65 (49.75–78)	66 (55–78)	n.s.
Inpatients	[% (*n*/total)]	79.4 (135/170)	82.8 (120/145)	81 (255/315)	n.s.
Outpatients	[% (*n*/total)]	20.6 (35/170)	17.2 (25/145)	19 (60/315)	
Length of hospital stay [days]	[median (IQR)]	13 (7–24.5)	10 (4–20)	12 (6–22)	n.s.
**Comorbidities and risk factors**					
Obstructive lung disease [OLD]	[% (*n*/total)]	22.6 (38/168)	31.2 (44/141)	26.5 (82/309)	n.s.
Lung transplant	[% (*n*/total)]	4.7 (8/170)	0 (0/142)	2.6 (8/309)	**0.009**
Chronic kidney failure	[% (*n*/total)]	39.9 (67/159)	26.4 (37/140)	33.8 (104/308)	**0.013**
Heart failure	[% (*n*/total)]	19.0 (32/168)	17.0 (24/141)	18.1 (56/309)	n.s.
Arterial hypertension	[% (*n*/total)]	58.9 (99/168)	57 (81/142)	58.1 (180/310)	n.s.
Coronary heart disease	[% (*n*/total)]	16.7 (28/168)	14.9 (21/141)	15.9 (49/309)	n.s.
Diabetes	[% (*n*/total)]	31.5 (53/168)	24.8 (35/141)	28.5 (88/309)	n.s.
Immunosuppression	[% (*n*/total)]	36.3 (61/168)	25.4 (36/142)	31.3 (97/310)	**0.038**
Malignancy	[% (*n*/total)]	35.7 (60/168)	28.2 (40/142)	32.3 (100/310)	n.s.
Solid	[% (*n*/total)]	11.9 (20/168)	4.9 (7(142)	8.7 (27/310)	n.s.
Hematologic	[% (*n*/total)]	23.2 (39/168)	22.5 (32/142)	22.9 (71/310)	
Solid and hematologic	[% (*n*/total)]	0.6 (1/168)	0.7 (1/142)	0.6 (2/310)	
CCI	[median (IQR)]	5 (4–7)	5 (3–7)	5 (3–7)	n.s.
**Clinical presentation and features**					
Fever	[% (*n*/total)]	24.1 (38/158)	35.8 (43/120)	29.1 (81/178)	**0.032**
Newly reported dyspnea	[% (*n*/total)]	43.5 (67/154)	48.4 (59/122)	45.7 (126/276)	n.s.
URTI	[% (*n*/total)]	35.4 (28/79)	34.0 (33/97)	34.7 (61/176)	n.s.
LRTI	[% (*n*/total)]	52.5 (83/158)	77.8 (84/108)	62.8 (167/266)	**<0.001**
Bronchitis	[% (*n*/total)]	7.6 (12/158)	10.3 (11/107)	8.7 (23/265)	n.s.
Pneumonia	[% (*n*/total)]	33.3 (53/159)	48.1 (52/108)	39.3 (105/267)	**0.015**
Exacerbation of OLD	[% (*n*/total)]	13.8 (22/159)	26.9 (29/108)	19.1 (51/167)	**0.008**
ICU stay	[% (*n*/total)]	25.9 (44/170)	21.1 (30/142)	23.7 (74/312)	n.s.
Length of ICU stay [days]	[median (IQR)]	3 (1–10)	5 (2.75–12.5)	3.5 (2–10)	n.s.
Ventilatory support	[% (*n*/total)]	15.3 (26/170)	23.2 (33/142)	18.9 (59/312)	n.s.
None *	[% (*n*/total)]	84.7 (144/170)	76.8 (109/142)	81.1 (253/312)	n.s.
HFNC	[% (*n*/total)]	0.6 (1/170)	1.4 (2/142)	1.0 (2/312)	
Non-invasive	[% (*n*/total)]	7.1 (12/170)	9.2 (13/142)	8.0 (25/312)	
Invasive	[% (*n*/total)]	7.6 (13/170)	12.7 (18/142)	9.9 (31/312)	
Administration of bronchodilators	[% (*n*/total)]	16.9 (28/166)	30.9 (42/136)	23.2 (70/302)	**0.004**
Syst. prednisolone administration	[% (*n*/total)]	14.9 (25/168)	22.0 (29/132)	18.0 (54/300)	n.s.
Co-infections	[% (*n*/total)]	21.2 (36/170)	27.3 (39/143)	24.0 (75/313)	n.s.
Bacterial	[% (*n*/total)]	5.9 (10/170)	11.9 (17/143)	8.6 (27/313)	n.s.
Viral	[% (*n*/total)]	11.2 (19/170)	9.8 (14/143)	10.5 (33/313)	
Fungal	[% (*n*/total)]	1.8 (3/170)	0.7 (1/143)	1.3 (4/313)	
Combined	[% (*n*/total)]	2.4 (4/170)	5.6 (8/143)	3.8 (12/313)	
Mortality	[% (*n*/total)]	6.5 (11/170)	12 (17/142)	9 (28/312)	n.s.

Analyzed categories are displayed in the column to the left and are either given as frequencies (%) or as the median and interquartile range (median (IQR)). (n/total) indicates the respective cases for the total amount of available data. The *p*-values of the chi-square tests for the contingency tables, including all subcategories, are indicated. All significant *p*-values for the post hoc pairwise analysis are given in the corresponding paragraph. The Mann–Whitney U test was performed to compare continuous variables. CCI, Charlson comorbidity index; HFNC, high-flow nasal cannula; ICU, intensive care unit; LRTI, lower respiratory tract infection; n.s., not significant; OLD, obstructive lung disease; syst., systemic; URTI, upper respiratory tract infection; * including low-flow oxygen via nasal cannula.

**Table 3:** Amino acid changes in RSV-A isolates.

**AA position**	9	10	12	13	20	65	103	105	113	114	122	127	276	334	377	384	543	547
**Subunit**	F1	F1	F1	F1	F1	F1	F1	F1					F2	F2	F2	F2	F2	F2
**Region**	SP	SP	SP	SP	SP				p27	p27	p27	P27					CT	CT
**Antigenic sites**						Ø/II							II/III		I/III	I		
**GA2.3.5 consensus**	**N**	**A**	**T**	**T**	**L**	**K**	**A**	**S**	**R**	**F**	**T**	**V**	**S**	**L**	**S**	**I**	**A**	**L**
16 strains *	.	.	.	.	.	.	.	.	.	.	.	.	.	.	.	.	.	.
RSVA/Leipzig/2017/1	.	.	.	.	.	.	.	N	.	.	.	.	.	.	.	.	.	.
RSVA/Leipzig/2018/2	.	.	.	.	.	.	.	N	.	.	.	.	.	.	.	.	.	.
RSVA/Leipzig/2018/3	.	.	.	.	.	.	.	N	.	.	.	.	.	.	.	.	.	I
RSVA/Leipzig/2018/5	.	.	.	.	.	.	.	N	.	.	.	.	.	.	.	.	.	I
RSVA/Leipzig/2018/9	.	.	.	.	.	.	.	.	.	.	.	.	.	.	.	T	.	.
RSVA/Leipzig/2018/10	.	.	.	.	.	.	.	N	.	.	.	.	.	.	.	.	.	.
RSVA/Leipzig/2018/11	.	.	.	.	.	.	.	N	.	.	.	.	.	.	.	.	.	.
RSVA/Leipzig/2018/12	.	.	.	.	.	.	.	.	.	.	.	A	.	.	.	.	.	.
RSVA/Leipzig/2018/14	.	.	.	.	.	.	.	N	.	.	.	.	.	.	.	.	.	.
RSVA/Leipzig/2018/17	.	.	.	.	.	.	.	N	.	.	.	.	.	.	.	.	.	.
RSVA/Leipzig/2018/15	.	.	.	.	.	.	.	.	.	.	.	.	N	.	.	.	.	.
RSVA/Leipzig/2018/18	.	.	.	.	.	.	.	.	.	.	.	.	N	.	.	.	.	.
RSVA/Leipzig/2018/16	.	.	.	.	.	.	.	.	.	.	.	.	N	.	.	.	.	.
RSVA/Leipzig/2019/3	.	.	.	.	.	.	.	.	.	.	.	.	N	.	.	.	.	.
RSVA/Leipzig/2019/4	.	.	.	.	.	.	.	.	.	.	.	.	N	.	.	.	.	.
RSVA/Leipzig/2019/5	.	.	.	.	.	.	.	.	.	.	.	.	N	.	.	.	.	.
RSVA/Leipzig/2019/8	.	.	.	.	.	.	.	.	.	.	.	.	N	.	.	.	.	.
RSVA/Leipzig/2019/10	.	.	.	.	.	.	.	.	.	.	.	.	.	.	.	.	T	.
RSVA/Leipzig/2019/11	.	.	.	.	.	.	.	.	.	.	.	.	N	.	.	.	.	.
RSVA/Leipzig/2019/14	.	.	.	.	.	.	.	.	.	.	.	.	N	.	.	.	.	.
RSVA/Leipzig/2019/20	.	.	I	.	.	.	.	.	.	.	.	.	N	.	.	.	.	.
RSVA/Leipzig/2019/16	.	.	.	.	.	.	.	.	.	.	.	.	N	.	.	.	.	.
RSVA/Leipzig/2020/10	.	.	.	.	.	.	.	.	.	S	.	.	.	I	.	.	.	.
RSVA/Leipzig/2020/16	D	.	.	A	.	.	.	.	.	.	.	.	.	.	.	.	.	.
RSVA/Leipzig/2021/11	.	.	I	.	.	.	.	.	.	.	.	.	.	.	.	.	.	.
RSVA/Leipzig/2021/18	.	.	.	.	.	.	.	.	.	.	.	.	.	.	N	.	.	.
RSVA/Leipzig/2021/47	.	.	I	.	.	.	.	.	.	.	.	.	.	.	.	.	.	.
RSVA/Leipzig/2021/67	.	.	I	.	F	R	.	.	.	.	.	.	N	.	.	.	.	.
RSVA/Leipzig/2021/73	.	.	.	.	.	.	.	.	.	.	.	.	.	.	N	.	.	.
RSVA/Leipzig/2021/123	.	.	I	.	F	.	.	.	.	.	.	.	N	.	.	.	.	.
RSVA/Leipzig/2021/129	.	.	.	.	.	.	.	.	I	.	.	.	.	.	N	.	.	.
RSVA/Leipzig/2022/1	.	.	.	.	.	.	.	.	I	.	.	.	.	.	N	.	.	.
RSVA/Leipzig/2022/6	.	V	I	.	F	.	.	.	.	.	.	.	.	.	.	.	.	.
RSVA/Leipzig/2022/7	.	V	I	.	F	.	.	.	.	.	.	.	.	.	.	.	.	.
RSVA/Leipzig/2022/8	.	.	I	.	F	.	.	.	.	.	.	.	.	.	.	.	.	.
RSVA/Leipzig/2022/9	.	.	I	.	F	.	.	.	.	.	.	.	.	.	.	.	.	.
RSVA/Leipzig/2022/10	.	V	I	.	F	.	.	.	.	.	.	.	.	.	.	.	.	.
RSVA/Leipzig/2022/11	.	V	I	.	F	.	.	.	.	.	.	.	.	.	.	.	.	.
RSVA/Leipzig/2022/1	.	.	.	.	.	.	T	.	.	.	A	.	.	.	.	.	.	.
RSVA/Leipzig/2022/2	.	.	.	.	.	.	T	.	.	.	A	.	.	.	.	.	.	.
RSVA/Leipzig/2022/3	.	V	I	.	F	.	.	.	.	.	.	.	.	.	.	.	.	.

Amino acids (AA) are numbered with regard to the F0 protein. Additionally, the F1 and F2 subunits are indicated. The consensus amino acid residues to which the isolates were compared were derived from the GA2.3.5 consensus sequence. The AA changes were mapped to the following regions or antigenic sites: SP, signal peptide; p27, 27 amino acid fragments; CT, cytoplasmatic tail; Ø, site Ø; I–III, site I to III. * Sixteen strains that were identical at the amino acid level are depicted as a pool and contain isolates of the following seasons: two strains for 2017/2018, ten strains for 2019/2020, four strains for 2021/22.

**Table 4:** Amino acid changes in RSV-B isolates.

**AA position**	9	12	22	42	64	108	113	116	123	157	190	191	206	209	211	273	276	295	389	463	477	507	508	514	527
**Subunit**	F1	F1	F1	F1	F1	F1				F2	F2	F2	F2	F2	F2	F2	F2	F2	F2	F2	F2	F2	F2	F2	F2
**Region**	SP	SP	SP				p27	p27	p27	A	A	A										B	B	B	CT
**Antigenic sites**				I	Ø							V	Ø	Ø	Ø	II	I/II	Ø	I						
**GB5.0.5a consensus**	**S**	**F**	**L**	**R**	**I**	**R**	**Q**	**N**	**K**	**V**	**S**	**K**	**I**	**Q**	**S**	**L**	**S**	**E**	**S**	**E**	**Y**	**R**	**R**	**H**	**I**
2 strains ^a^	.	.	.	.	.	.	.	.	.	.	.	.	.	.	.	.	.	.	.	.	.	.	.	.	.
25 strains ^b^	.	.	.	.	.	.	.	.	.	.	.	R	M	R	.	.	.	.	.	.	.	.	.	.	.
RSVB/Leipzig/2018/3	.	.	.	.	.	.	.	.	.	.	.	R	M	R	.	.	.	D	.	.	.	.	.	.	.
RSVB/Leipzig/2018/13	.	.	.	.	.	.	.	.	.	.	.	R	M	R	.	.	.	.	.	.	.	.	.	.	V
RSVB/Leipzig/2018/15	.	.	.	.	.	.	.	.	.	.	.	R	M	R	.	.	N	.	.	.	.	.	.	.	.
RSVB/Leipzig/2019/7	.	.	.	.	.	.	.	.	.	.	.	R	M	R	.	.	N	.	.	.	.	.	.	.	.
RSVB/Leipzig/2019/8	.	L	.	.	.	.	.	.	.	.	.	R	M	R	.	.	N	.	.	D	.	.	.	.	.
RSVB/Leipzig/2019/11	.	L	.	.	.	.	.	.	.	.	.	R	M	R	.	.	N	.	.	.	.	.	.	.	.
RSVB/Leipzig/2019/12	.	L	F	.	.	.	.	.	.	.	N	R	M	R	.	.	N	.	.	D	.	.	.	.	.
RSVB/Leipzig/2021/19	.	.	.	.	.	.	.	.	.	A	N	R	M	R	N	.	.	.	P	.	.	.	.	.	.
21 strains ^c^	.	.	.	.	.	.	.	.	.	.	N	R	M	R	N	.	.	.	P	.	.	.	.	.	.
RSVB/Leipzig/2022/3	.	.	.	K	.	.	.	.	.	.	N	R	M	R	N	.	.	.	P	.	.	.	.	.	.
RSVB/Leipzig/2022/4	.	.	.	.	.	.	.	.	R	.	.	R	M	R	.	.	.	.	.	.	.	.	.	.	.
RSVB/Leipzig/2022/5	.	.	.	.	.	.	.	.	R	.	.	R	M	R	.	.	.	.	.	.	.	.	.	.	.
RSVB/Leipzig/2022/6	.	.	.	.	.	.	.	.	.	.	N	R	M	R	N	.	.	.	P	.	.	H	.	R	.
RSVB/Leipzig/2022/7	.	.	.	.	.	.	.	.	R	.	.	R	M	R	.	.	.	.	.	.	.	.	.	.	.
RSVB/Leipzig/2022/8	.	.	.	.	.	.	.	.	R	.	.	R	M	R	.	.	.	.	.	.	.	.	.	.	.
RSVB/Leipzig/2022/9	.	.	.	K	.	.	.	.	.	.	N	R	M	R	N	.	.	.	P	.	.	.	.	.	.
RSVB/Leipzig/2022/10	.	.	.	K	V	.	.	.	.	.	N	R	M	R	N	.	.	.	P	.	.	.	.	.	.
RSVB/Leipzig/2022/11	.	.	.	.	.	.	.	S	.	.	N	R	M	R	N	.	.	.	P	.	.	.	.	.	.
RSVB/Leipzig/2022/14	.	.	.	K	.	.	.	.	.	.	N	R	M	R	N	.	.	.	P	.	.	.	.	.	.
RSVB/Leipzig/2022/15	.	.	.	K	.	.	.	.	.	.	N	R	M	R	N	.	.	.	P	.	.	.	.	.	.
RSVB/Leipzig/2022/16	N	.	.	K	.	.	.	.	.	.	N	R	M	R	N	.	.	.	P	.	.	.	.	.	.
RSVB/Leipzig/2022/17	.	.	.	.	.	.	.	.	.	.	N	R	M	.	N	.	.	.	P	.	.	.	.	.	.
RSVB/Leipzig/2022/18	.	.	.	.	.	.	R	.	.	.	N	R	M	R	N	.	.	.	P	.	.	.	.	.	.
RSVB/Leipzig/2022/19	.	.	.	K	.	.	.	.	.	.	N	R	M	R	N	.	.	.	P	.	.	.	.	.	.
RSVB/Leipzig/2022/20	.	.	.	.	.	.	.	.	R	.	.	R	M	R	.	.	.	.	.	.	.	.	.	.	.
RSVB/Leipzig/2022/21	.	.	.	.	.	.	.	.	.	.	N	R	M	R	N	.	.	.	P	.	.	.	.	.	.
RSVB/Leipzig/2022/22	.	.	.	K	.	K	.	.	.	.	N	R	M	R	N	.	.	.	P	.	.	.	.	.	.
RSVB/Leipzig/2023/1	.	.	.	K	.	.	.	.	.	.	N	R	M	R	N	.	.	.	P	.	.	.	.	.	.
RSVB/Leipzig/2023/3	.	.	.	.	.	.	.	.	.	.	N	R	M	R	N	I	.	.	P	.	.	.	.	.	.
RSVB/Leipzig/2023/5	.	.	.	K	.	.	.	.	.	.	N	R	M	R	N	.	.	.	P	.	.	.	.	.	.
RSVB/Leipzig/2023/6	.	.	.	.	.	.	.	S	.	.	N	R	M	R	N	.	.	.	P	.	.	.	.	.	.
RSVB/Leipzig/2023/7	.	.	.	K	.	.	.	.	.	.	N	R	M	R	N	.	.	.	P	.	.	.	.	.	.
RSVB/Leipzig/2023/10	.	.	.	.	.	.	.	.	.	.	N	R	M	R	N	.	.	.	P	.	.	H	.	R	.
RSVB/Leipzig/2023/11	.	.	.	.	.	.	.	.	R	.	.	R	M	R	.	.	.	.	.	.	.	.	.	.	.
RSVB/Leipzig/2023/12	.	.	.	.	.	.	.	.	.	.	N	R	M	R	N	.	.	.	P	.	H	.	.	.	.
RSVB/Leipzig/2023/13	.	.	.	.	.	.	.	S	.	.	N	R	M	R	N	.	.	.	P	.	.	.	.	.	.
RSVB/Leipzig/2023/16	.	.	.	.	.	.	.	.	.	.	N	R	M	R	N	.	.	.	P	.	H	.	.	.	.
RSVB/Leipzig/2023/17	.	.	.	.	.	.	.	.	R	.	.	R	M	R	.	.	.	.	.	.	.	.	.	.	.
RSVB/Leipzig/2023/18	.	.	.	K	.	.	.	.	.	.	N	R	M	R	N	.	.	.	P	.	.	.	.	.	.
RSVB/Leipzig/2023/19	.	.	.	.	.	.	.	.	R	.	.	R	M	R	.	.	.	.	.	.	.	.	.	.	.
RSVB/Leipzig/2023/20	.	.	.	.	.	.	.	.	R	.	.	R	M	R	.	.	.	.	.	.	.	.	.	.	.
RSVB/Leipzig/2023/21	.	.	.	.	.	.	.	.	R	.	.	R	M	R	.	.	.	.	.	.	.	.	.	.	.
RSVB/Leipzig/2023/24	.	.	.	.	.	.	.	.	R	.	.	R	M	R	.	.	.	.	.	.	.	.	.	.	.
RSVB/Leipzig/2023/25	.	I	.	.	.	.	.	.	.	.	N	R	M	R	N	.	.	.	P	.	.	.	K	.	.

Amino acids (AA) are numbered with regard to the F0 protein. Additionally, the F1 and F2 subunits are indicated. The consensus amino acid residues to which the isolates were compared were derived from the GB5.0.5a consensus sequence. The AA changes were mapped to the following regions or antigenic sites: SP, signal peptide; p27, 27 amino acid fragments; A, heptad repeat A; B, heptad repeat B; CT, cytoplasmatic tail; Ø, site Ø; I, II, V, sites I, II, V. Strains that were identical at the amino acid level are depicted as a pool and contain isolates of the following seasons: ^a^ two strains for 2017/2018; ^b^ seven for 2017/2018, five for 2018/2019, ten for 2019/2020, three for 2021/2022; ^c^ nine for 2021/2022, 12 for 2022/2023.

## Data Availability

Identified sequences were submitted to GenBank (accession No. PP084746-PP084890 and PP354071-PP354073).

## Supplementary Materials

The following supporting information can be downloaded at https://www.mdpi.com/article/10.3390/v16060943/s1, Table S1: Primers used for RSV (F gene) sequencing, Table S2: Reagent composition for RSV-A sequencing (first PCR), Table S3: Reagent composition for RSV-B sequencing (first PCR), Table S4: Cycling conditions for RSV sequencing (first PCR), Table S5: Reagent composition for RSV-A sequencing (nested PCR), Table S6: Reagent composition for RSV-B sequencing (nested PCR), Table S7: Cycling conditions for RSV sequencing (nested PCR), Table S8: RSV-A reference sequences by Goya et al., Table S9: RSV-B reference sequences by Goya et al., Table S10: Amino acid residues of antigenic sites Ø–V, Table S11: Co-infecting pathogens, Table S12: Study population and clinical features of RSV cases in the 2021/2022 and 2022/2023 seasons, Figure S1: Molecular phylogenetic analysis of the RSV-A F gene by the maximum likelihood method, Figure S2: Molecular phylogenetic analysis of the RSV-B F gene by the maximum likelihood method.
