# Supplementary material for: Respiratory Syncytial Virus in Adult Patients at a Tertiary Care Hospital in Germany: Clinical Features and Molecular Epidemiology of the Fusion Protein in the Severe Respiratory Season of 2022/2023
Source: Viruses. 2024 Jun 12;16(6):943. doi: 10.3390/v16060943 (PMC11209376; doi:10.3390/v16060943)
Supplement: Supplementary file 1 [file viruses-16-00943-s001.zip › viruses-3029051-supplementary.pdf]

# Respiratory syncytial virus in adult patients at a tertiary care hospital in Germany: clinical features and molecular epidemiology of the fusion protein in the severe respiratory season of 2022/2023

Mario Hönemann, Melanie Maier, Armin Frille, Stephanie Thiem, Sandra Bergs, Thomas C. Williams, Vicente Mas, Christoph Lübbert, and Corinna Pietsch

## Supplementary Material

**Table S1.** Primers used for RSV (F gene) sequencing.

| Primer | Sequence (5′ to 3′) |
|---|---|
| RSV-A-F-FNA | TCCACTCAACCACCTCCGAA |
| RSV-A-F-FNI1 | ACATCCGAGTACCTATCACAATCT |
| RSV-A-F-FNI2 | AGACAGCAAAGTTACTCTATCATGTC |
| RSV-A-F-RNA | TCCAACTCTGCAGCTCCACT |
| RSV-A-F-RNI1 | TACCATCCTCTGTCGGTTCTTG |
| RSV-A-F-RNI2 | TGTCTTACAAGCAGTGCATGGG |
| RSV-B-F-FNA | ACACCAGCACCTCACAATCCA |
| RSV-B-F-FNI1 | CACCCAACTCCACACAAACAC |
| RSV-B-F-FNI2 | CAGTTATAGAATTCCAGCAGAAGAAC |
| RSV-B-F-RNA | TGCTCTTGTCCATTGACTTGAGTAT |
| RSV-B-F-RNI1 | AGCAAGGTGTATCAATTACACCATAGAT |
| RSV-B-F-RNI2 | GGCCATTCAAAGTAATTATGACTGTAG |

Primer concentration: 10 pmol/µl. The primers were designed for the current study.

**Table S2.** Reagent composition for RSV-A sequencing (first PCR).

| | Concentration | Volume [µl] | Final concentration |
|---|---|---|---|
| **$H_20$** | | 20 | |
| **5xBuffer** | 5x (+12,5mM $MgCl_2$) | 10 | 1x (+2,5mM $MgCl_2$) |
| **Primer 1** | 10 pmol/µl | 3 | 30 pmol |
| **Primer 2** | 10 pmol/µl | 3 | 30 pmol |
| **dNTP's** | 10 mM each | 1 | 200 µM |
| **Enzyme-Mix** | | 2 | |
| **RNase Inhibitor** | 40 Units/µl | 1 | 40 Units |
| **RNA** | | 10 | |

Reagent concentrations and volumes used for RSV-A sequencing (F gene). The QIAGEN® OneStep RT-PCR Kit (Cat.No.210212) was used. Two separate nested PCRs with different primer sets were performed for the amplification of two fragments: RSV-A-F-FNA and RSV-A-F-RNI1 (set 1) and RSV-A-F-FNI2 and RSV-A-F-RNA (set 2).

**Table S3.** Reagent composition for RSV-B sequencing (first PCR).

| | Concentration | Volume [µl] | Final concentration |
|---|---|---|---|
| **H20** | | 20 | |
| **5xBuffer** | 5x (+12,5mM $MgCl_2$) | 10 | 1x (+2,5mM $MgCl_2$) |
| **Primer 1** | 10 pmol/µl | 3 | 30 pmol |
| **Primer 2** | 10 pmol/µl | 3 | 30 pmol |
| **dNTP's** | 10 mM each | 1 | 200 µM |
| **Enzyme-Mix** | | 2 | |
| **RNase Inhibitor** | 40 Units/µl | 1 | 40 Units |
| **RNA** | | 10 | |

Reagent concentrations and volumes used for RSV-B sequencing (F Gene). The QIAGEN® OneStep RT-PCR Kit (Cat.No.210212) was used. Two separate nested PCRs with different primer sets were performed for the amplification of two fragments: RSV-B-F-FNA and RSV-B-F-RNI1 (set 1) and RSV-B-F-FNI2 and RSV-B-F-RNA (set 2).

**Table S4.** Cycling conditions for RSV-Sequencing (first PCR).

| Reaction | Temperature | Time | Cycles |
|---|---|---|---|
| **Reverse transcription** | 50°C | 30 min | 1 |
| **Enzyme activation** | 95°C | 15 min | 1 |
| **Denaturation** | 94°C | 1 min | 45 |
| **Annealing** | 56°C | 30 s | |
| **Elongation** | 72°C | 1 min 30 s | |
| **Final elongation** | 72°C | 5 min | 1 |
| **Cooling** | 4°C | forever | |

The amplification was performed with a GeneTouch Thermal Cycler BTC33BAS (GeneTouch Corp., Taoyuan City, Taiwan).

**Table S5.** Reagent composition for RSV-A sequencing (nested PCR).

| | Concentration | Volume [µl] | Final concentration |
|---|---|---|---|
| **$H_20$** | | 33,1 | |
| **10xBuffer,-$MgCl_2$** | 10x | 5 | 1x |
| **$MgCl_2$** | 50 mM | 1,5 | 1,5 mM |
| **Primer 1** | 10 pmol/µl | 2 | 20 pmol |
| **Primer 2** | 10 pmol/µl | 2 | 20 pmol |
| **dNTP's** | 10 mM each | 1 | 200 µM |
| **Platinum Taq** | 5 Units/µl | 0,4 | 2 Units |
| **First round PCR-product** | | 5 | |

Reagent concentrations and volumes used for RSV-A sequencing (F gene). The Invitrogen Platinum™ II Taq DNA Polymerase (Cat.No. 10966034) was used. Two separate nested PCRs with different primer sets were performed for the amplification of two fragments: RSV-A-F-FNI1 and RSV-A-F-RNI1 (set 1) and RSV-A-F-FNI2 and RSV-A-F-RNI2 (set 2).

**Table S6.** Reagent composition for RSV-B sequencing (nested PCR).

| | Concentration | Volume [µl] | Final concentration |
|---|---|---|---|
| **$H_20$** | | 33,1 | |
| **10xBuffer,-$MgCl_2$** | 10x | 5 | 1x |
| **$MgCl_2$** | 50 mM | 1,5 | 1,5 MM |
| **Primer 1** | 10 pmol/µl | 2 | 20 pmol |
| **Primer 2** | 10 pmol/µl | 2 | 20 pmol |
| **dNTP's** | 10 mM each | 1 | 200 µM |
| **Platinum Taq** | 5 Units/µl | 0,4 | 2 Units |
| **First round PCR-product** | | 5 | |

Reagent concentrations and volumes used for RSV-A sequencing (F gene). The Invitrogen Platinum™ II Taq DNA Polymerase (Cat.No. 10966034) was used. Two separate nested PCRs with different primer sets were performed for the amplification of two fragments: RSV-B-F-FNI1 and RSV-B-F-RNI1 (set 1) and RSV-B-F-FNI2 and RSV-B-F-RNI2 (set 2).

**Table S7.** Cycling conditions for Sanger Sequencing (nested PCR).

| Reaction | Temperature | Time | Cycles |
|---|---|---|---|
| **Enzyme activation** | 95°C | 1 min | 1 |
| **Denaturation** | 94°C | 30 s | 45 |
| **Annealing** | 56°C | 30 s | |
| **Elongation** | 72°C | 1 min 20 s | |
| **Final elongation** | 72°C | 5 min | 1 |
| **Cooling** | 4°C | forever | |

The amplification was performed on a GeneTouch Thermal Cycler BTC33BAS (GeneTouch Corp., Taoyuan City, Taiwan). The resulting PCR products were used for Sanger sequencing.

**Table S8.** RSV-A Reference Sequences Goya et al.

| | | | | | |
|---|---|---|---|---|---|
| GA1 | AY911262* | GA2.3.3 | KY654511 | GA3.0.0 | KU316149 |
| GA1 | JX198138 | GA2.3.3 | MF001041 | GA3.0.0 | MG642074 |
| GA1 | KJ723474 | GA2.3.3 | MF001047 | GA3.0.1 | KP258699 |
| GA1 | KU316165 | GA2.3.3 | MF001054 | GA3.0.1 | KU316133 |
| GA2 | MG642063 | GA2.3.4 | JF920053 | GA3.0.1 | MG642031 |
| GA2.1 | KJ723483 | GA2.3.4 | KC731483 | GA3.0.2 | KJ723465 |
| GA2.1 | KP258723 | GA2.3.4 | KJ672455 | GA3.0.2 | KP258701 |
| GA2.1 | KU316098 | GA2.3.4 | KJ672482 | GA3.0.2 | KP258726 |
| GA2.1 | MG642070 | GA2.3.4 | KP663728 | GA3.0.2 | KU316104 |
| GA2.2 | JF920062 | GA2.3.4 | KU950667 | GA3.0.2 | KU316161 |
| GA2.2 | JX069801 | GA2.3.4 | KX655658 | GA3.0.2 | KU316170 |
| GA2.2 | KJ723492 | GA2.3.4 | KX765920 | GA3.0.2 | MG642048 |
| GA2.2 | KP258700 | GA2.3.4 | KY460517 | GA3.0.3a | JQ901455 |
| GA2.2 | KP258743 | GA2.3.4 | KY654508 | GA3.0.3a | JX069802 |
| GA2.2 | KU316092 | GA2.3.4 | MF001051 | GA3.0.3a | KF826847 |
| GA2.2 | MG642030 | GA2.3.4 | MF001053 | GA3.0.3a | KM360090 |
| GA2.3.0 | JX069798 | GA2.3.5 | KJ672467 | GA3.0.3a | KY967364 |
| GA2.3.0 | KP119748 | GA2.3.5 | KJ672470 | GA3.0.4a | KF530260 |
| GA2.3.0 | KU316118 | GA2.3.5 | KT285064 | GA3.0.4a | KF826854 |
| GA2.3.0 | KU950573 | GA2.3.5 | KU950506 | GA3.0.4b | KF826826 |
| GA2.3.0 | MG642033 | GA2.3.5 | KU950531 | GA3.0.4b | KF826827 |
| GA2.3.1 | JQ901452 | GA2.3.5 | KU950540 | GA3.0.4b | KF826850 |
| GA2.3.1 | JX015480 | GA2.3.5 | KU950550 | GA3.0.4b | KF973333 |
| GA2.3.1 | KJ627305 | GA2.3.5 | KU950556 | GA3.0.5b | KF826832 |
| GA2.3.2b | JX015486 | GA2.3.5 | KU950560 | GA3.0.5b | KX765933 |
| GA2.3.2b | KJ627284 | GA2.3.5 | KU950596 | GA3.0.5b | MF001038 |
| GA2.3.2b | KJ627336 | GA2.3.5 | KU950650 | | |
| GA2.3.3 | JX015482 | GA2.3.5 | KU950651 | | |
| GA2.3.3 | JX015491 | GA2.3.5 | KU950670 | | |
| GA2.3.3 | JX015497 | GA2.3.5 | KU950692 | | |
| GA2.3.3 | KF826838 | GA2.3.5 | KX765917 | | |
| GA2.3.3 | KF826855 | GA2.3.5 | KX765932 | | |
| GA2.3.3 | KJ627256 | GA2.3.5 | KX765941 | | |
| GA2.3.3 | KJ627294 | GA2.3.5 | KX765954 | | |
| GA2.3.3 | KJ627320 | GA2.3.5 | KX765971 | | |
| GA2.3.3 | KJ627337 | GA2.3.5 | KX894807 | | |
| GA2.3.3 | KJ627338 | GA2.3.5 | KY654514 | | |
| GA2.3.3 | KJ627349 | GA2.3.5 | KY654518 | | |
| GA2.3.3 | KJ627370 | GA2.3.5 | KY883567 | | |
| GA2.3.3 | KJ939951 | GA2.3.5 | MG773271 | | |
| GA2.3.3 | KP317953 | GA3.0.0 | KJ723486 | | |
| GA2.3.3 | KX655662 | GA3.0.0 | KP258709 | | |
| GA2.3.3 | KX655672 | GA3.0.0 | KP258715 | | |
| GA2.3.3 | KX765958 | GA3.0.0 | KU316137 | | |

Accession numbers of proposed genotypes [16]. *This sequence was used as RSV-A prototype strain sequence for the rooting of the phylogenetic trees throughout the manuscript.

**Table S9.** RSV-B Reference Sequences Goya et al.

| | | | |
|---|---|---|---|
| GB1 | AY353550* | GB5.0.3 | MG431252 |
| GB1 | JX198143 | GB5.0.4a | KJ939929 |
| GB1 | KP258736 | GB5.0.4a | KJ939932 |
| GB2 | AF013254 | GB5.0.4a | KU950467 |
| GB2 | JX198165 | GB5.0.4a | KX655648 |
| GB2 | KP258712 | GB5.0.4a | KX655653 |
| GB2 | KU316127 | GB5.0.4a | KX765912 |
| GB2 | KU316173 | GB5.0.4a | KX765957 |
| GB2 | KU316175 | GB5.0.4a | KX765962 |
| GB2 | KU316181 | GB5.0.4a | KY249657 |
| GB2 | KU316182 | GB5.0.4a | KY249670 |
| GB2 | MG642036 | GB5.0.4a | KY249677 |
| GB2 | MG642043 | GB5.0.4a | KY883571 |
| GB3 | JX198147 | GB5.0.4b | JN032115 |
| GB3 | JX198166 | GB5.0.4b | JX576730 |
| GB4 | JX198160 | GB5.0.4b | JX576746 |
| GB4 | MG642062 | GB5.0.4b | JX576751 |
| GB5.0.0 | JX576760 | GB5.0.4b | KF826860 |
| GB5.0.0 | KF826853 | GB5.0.4b | KJ627285 |
| GB5.0.0 | KP258713 | GB5.0.4c | KJ627262 |
| GB5.0.0 | KP258724 | GB5.0.4c | KJ939928 |
| GB5.0.0 | KP317923 | GB5.0.4c | KP317928 |
| GB5.0.0 | KU316134 | GB5.0.4c | KU950477 |
| GB5.0.0 | KU316179 | GB5.0.4c | KU950588 |
| GB5.0.0 | MF185754 | GB5.0.4c | KX655649 |
| GB5.0.1 | JX576761 | GB5.0.4c | KX655654 |
| GB5.0.1 | JX576762 | GB5.0.4c | KX765949 |
| GB5.0.1 | KF826829 | GB5.0.4c | KY249658 |
| GB5.0.1 | KJ939919 | GB5.0.4c | MG431251 |
| GB5.0.1 | MF185752 | GB5.0.5a | KX765906 |
| GB5.0.2 | JX576742 | GB5.0.5a | KY249683 |
| GB5.0.2 | KF826843 | GB5.0.5a | KY684758 |
| GB5.0.2 | KF826845 | GB5.0.5a | MG773268 |
| GB5.0.2 | KJ627302 | GB5.0.5a | MG839547 |
| GB5.0.2 | KJ939926 | GB6 | MF185751 |
| GB5.0.2 | KU950484 | | |
| GB5.0.2 | KU950619 | | |
| GB5.0.2 | KX655669 | | |
| GB5.0.2 | KX765943 | | |
| GB5.0.2 | KY249662 | | |
| GB5.0.3 | JN032117 | | |
| GB5.0.3 | JX576744 | | |
| GB5.0.3 | KF826839 | | |
| GB5.0.3 | KU950458 | | |

Accession numbers of proposed genotypes [16]. *This sequence was used as RSV-B prototype strain sequence for the rooting of the phylogenetic trees throughout the manuscript.

**Table S10.** Amino acid residues of antigenic sites Ø – V.

| Antigenic site | Footprints | Residues [n] | Residues |
|---|---|---|---|
| Site Ø | 5C4 | 26 | 63, 64, 65, 66, 67, 68, 69, 77, 83, 168, 196, 197, 198, 200, 201, 202, 204, 205, 206, 207, 208, 209, 211, 212, 294, 295 |
| | D25 | 28 | 62, 63, 64, 65, 66, 67, 68, 69, 71, 72, 73, 74, 83, 197, 198, 200, 201, 202, 204, 205, 206, 207, 208, 209, 210, 211, 212, 216 |
| | 5C4 + D25 | 33 | 62, 63, 64, 65, 66, 67, 68, 69, 71, 72, 73, 74, 77, 83, 168, 196, 197, 198, 200, 201, 202, 204, 205, 206, 207, 208, 209, 210, 211, 212, 216, 294, 295 |
| Site I | ADI-14349 | 20 | 31, 32, 33, 34, 35, 40, 42, 43, 312, 313, 314, 344, 377, 378, 380, 381, 383, 384, 389, 390 |
| Site II | Motavizumab | 20 | 65, 95, 176, 255, 258, 259, 261, 262, 263, 267, 268, 269, 271, 272, 275, 276, 309, 310, 363, 364 |
| Site III | MPE8 | 50 | 45, 50, 51, 52, 53, 54, 150, 178, 180, 185, 186, 187, 188, 262, 263, 264, 265, 266, 267, 268, 269, 270, 271, 272, 273, 276, 277, 305, 306, 307, 309, 310, 311, 312, 344, 345, 346, 347, 364, 377, 425, 427, 428, 429, 430, 431, 448, 449, 456, 458 |
| Site IV | 101F | 30 | 50, 416, 418, 419, 420, 421, 422, 423, 425, 426, 428, 429, 430, 431, 432, 433, 434, 435, 436, 437, 440, 446, 450, 451, 452, 453, 454, 455, 456, 457 |
| Site V | hRSV90 | 21 | 169, 170, 171, 172, 173, 174, 175, 176, 177, 178, 188, 191, 194, 196, 197, 200, 201, 226, 262, 263, 271 |

The antigenic sites were investigated as described in Mas et al. [25]. Residues that are located on the neighboring protomer are underlined.

**Table S11.** Co-infecting pathogens.

| Bacteria | *n* | Viruses | *n* | Fungi | *n* |
|---|---|---|---|---|---|
| *Pseudomonas aeruginosa* | 9 | Influenza A H3N2 | 16 | *Aspergillus fumigatus* | 2 |
| *Klebsiella pneumoniae* | 8 | SARS-CoV-2 | 12 | *Aspergillus niger* | 2 |
| *Streptococcus pneumoniae* | 6 | Parainfluenzavirus Type 3 | 6 | *Pneumocystis jirovecii* | 2 |
| *Staphylococcus aureus* | 5 | Rhinovirus | 5 | | |
| *Escherichia coli* | 3 | Metapneumovirus | 3 | | |
| *Serratia marcescens* | 3 | CMV | 2 | | |
| *Haemophilus influenzae* | 2 | Coronavirus OC43 | 2 | | |
| *Enterobacter cloacae* | 2 | Influenza B | 2 | | |
| *Chlamydophila pneumoniae* | 1 | VZV | 1 | | |
| *Enterococcus faecium* | 1 | Influenza A H1N1 | 1 | | |
| *Haemophilus parahaemolyticus* | 1 | Adenovirus | 1 | | |
| *Proteus mirabilis* | 1 | Parainfluenzavirus Type 1 | 1 | | |
| *Raoultella ornithinolytica* | 1 | HSV 1 | 1 | | |
| *Stenotrophomonas maltophilia* | 1 | Dengue Virus | 1 | | |

Pathogens detected by type, with n showing their frequencies of detection. The co-infections included cases with detections of more than one pathogen.

**Table S12.** Study population and clinical features of season 2021/2022 and season 2022/2023 RSV cases.

| | | 2021/2022 | 2022/2023 | total | *p*-value |
|---|---|---|---|---|---|
| **Study population** | | | | | |
| Female | [% (*n*/total)] | 42.3 (11/26) | 47.9 (70/146) | 47.1 (81/172) | n.s. |
| Male | [% (*n*/total)] | 57.6 (15/26) | 52.1 (76/146) | 52.9 (91/172) | |
| Age [years] | [median (IQR)] | 49.5 (31.5 – 64.25) | 65 (49.75 – 78) | 64 (46 – 75.75) | **<0.001** |
| Inpatients | [% (*n*/total)] | 76.0 (19/25) | 82.8 (120/145) | 81.8 (139/170) | n.s. |
| Outpatients | [% (*n*/total)] | 24.0 (6/25) | 17.2 (25/145) | 18.2 (31/170) | |
| Length of hospital stay [days] | [median (IQR)] | 12 (6.5 – 28) | 10 (4 – 20) | 10.5 (4.74 – 19.25) | n.s. |
| **Comorbidities and risk factors** | | | | | |
| Obstructive lung disease [OLD] | [% (*n*/total)] | 20.8 (5/24) | 31.2 (44/141) | 29.7 (49/165) | n.s. |
| Lung transplant | [% (*n*/total)] | 3.8 (1/26) | 0 (0/142) | 0.6 (1/168) | **0.019** |
| Chronic kidney failure | [% (*n*/total)] | 20.8 (5/24) | 26.4 (37/140) | 25.6 (42/164) | n.s. |
| Heart failure | [% (*n*/total)] | 8.3 (2/24) | 17.0 (24/141) | 15.8 (26/165) | n.s. |
| Arterial hypertension | [% (*n*/total)] | 37.5 (9/24) | 57.0 (81/142) | 54.2 (90/166) | n.s. |
| Coronary heart disease | [% (*n*/total)] | 8.3 (2/24) | 14.9 (21/141) | 13.9 (23/165) | n.s. |
| Diabetes | [% (*n*/total)] | 29.2 (7/24) | 24.8 (35/141) | 25.5 (42/165) | n.s. |
| Immunosuppression | [% (*n*/total)] | 50.0 (12/24) | 25.4 (36/142) | 28.9 (48/166) | **0.014** |
| Malignancy | [% (*n*/total)] | 29.2 (7/24) | 28.2 (40/142) | 28.3 (47/166) | n.s. |
| Solid | [% (*n*/total)] | 0 (0/24) | 4.9 (7(142) | 4.2 (7/166) | n.s. |
| Haematologic | [% (*n*/total)] | 20.8 (5/24) | 22.5 (32/142) | 22.3 (37/166) | |
| Solid and haematologic | [% (*n*/total)] | 8.3 (2/24) | 0.7 (1/142) | 1.8 (3/166) | |
| CCI | [median (IQR)] | 3.5 (1.75 – 5.25) | 5 (3 – 7) | 5 (3 – 6) | n.s. |
| **Clinical presentation and features** | | | | | |
| Fever | [% (*n*/total)] | 21.0 (4/19) | 35.8 (43/120) | 33.8 (47/139) | n.s. |
| Newly reported dyspnea | [% (*n*/total)] | 36.8 (7/19) | 48.4 (59/122) | 46.8 (66/141) | n.s. |
| URTI | [% (*n*/total)] | 33.3 (4/12) | 34.0 (33/97) | 33.9 (37/109) | n.s. |
| LRTI | [% (*n*/total)] | 63.2 (12/19) | 77.8 (84/108) | 75.6 (96/127) | n.s. |
| Bronchitis | [% (*n*/total)] | 15.8 (3/19) | 10.3 (11/107) | 11.1 (14/126) | n.s. |
| Pneumonia | [% (*n*/total)] | 42.1 (8/19) | 48.1 (52/108) | 47.2 (60/127) | n.s. |
| Exacerbation of OLD | [% (*n*/total)] | 10.5 (2/19) | 26.9 (29/108) | 24.4 (31/127) | n.s. |
| ICU stay | [% (*n*/total)] | 19.2 (5/26) | 21.1 (30/142) | 20.8 (35/168) | n.s. |
| Length of ICU stay [days] | [median (IQR)] | 2 (1.5 – 7) | 5 (2.75 – 12.5) | 4 (2 -10) | n.s. |
| Ventilatory support | [% (*n*/total)] | 11.5 (3/26) | 23.2 (33/142) | 21.7 (36/166) | n.s. |
| None* | [% (*n*/total)] | 88.5 (23/26) | 76.8 (109/142) | 79.5 (132/166) | n.s. |
| HFNC | [% (*n*/total)] | 0 (0/26) | 1.4 (2/142) | 1.2 (2/166) | |
| Non-invasive | [% (*n*/total)] | 3.8 (1/26) | 9.2 (13/142) | 8.4 (14/166) | |
| Invasive | [% (*n*/total)] | 7.7 (2/26) | 12.7 (18/142) | 12.0 (20/166) | |
| Administration of bronchodilators | [% (*n*/total)] | 12.5 (3/24) | 30.9 (42/136) | 29.8 (45/152) | n.s. |
| Syst. Prednisolone administration | [% (*n*/total)] | 12.5 (3/24) | 22 (29/132) | 20.5 (32/156) | n.s. |
| Co-infections | [% (*n*/total)] | 19.2 (5/26) | 27.3 (39/143) | 26.0 (44/169) | n.s. |
| Bacterial | [% (*n*/total)] | 7.7 (2/26) | 11.2 (16/143) | 10.7 (18/169) | n.s. |
| Viral | [% (*n*/total)] | 11.5 (3/26) | 10.5 (15/143) | 10.7 (18/169) | |
| Fungal | [% (*n*/total)] | 0 (0/26) | 0.7 (1/143) | 0.6 (1/169) | |
| Combined | [% (*n*/total)] | 0 (0/26) | 5.6 (8/143) | 4.7 (8/169) | |
| Mortality | [% (*n*/total)] | 3.8 (1/26) | 12.0 (17/142) | 10.8 (18/166) | n.s. |

Analyzed categories are displayed in the column to the left and are either given as frequencies (%) or as median and interquartile range (median (IQR)). (n/total) indicates the respective cases for the total amount of available data. The p-values of the chi-square tests for the contingency tables including all subcategories are indicated. The Mann–Whitney U test was performed to compare continuous variables. CCI, Charlson comorbidity index; HFNC, high-flow nasal cannula; ICU, intensive care unit; LRTI, lower respiratory tract infection; n.s., not significant; OLD, obstructive lung disease; syst., systemic; URTI, upper respiratory tract infection; *including low flow-oxygen via nasal cannula.

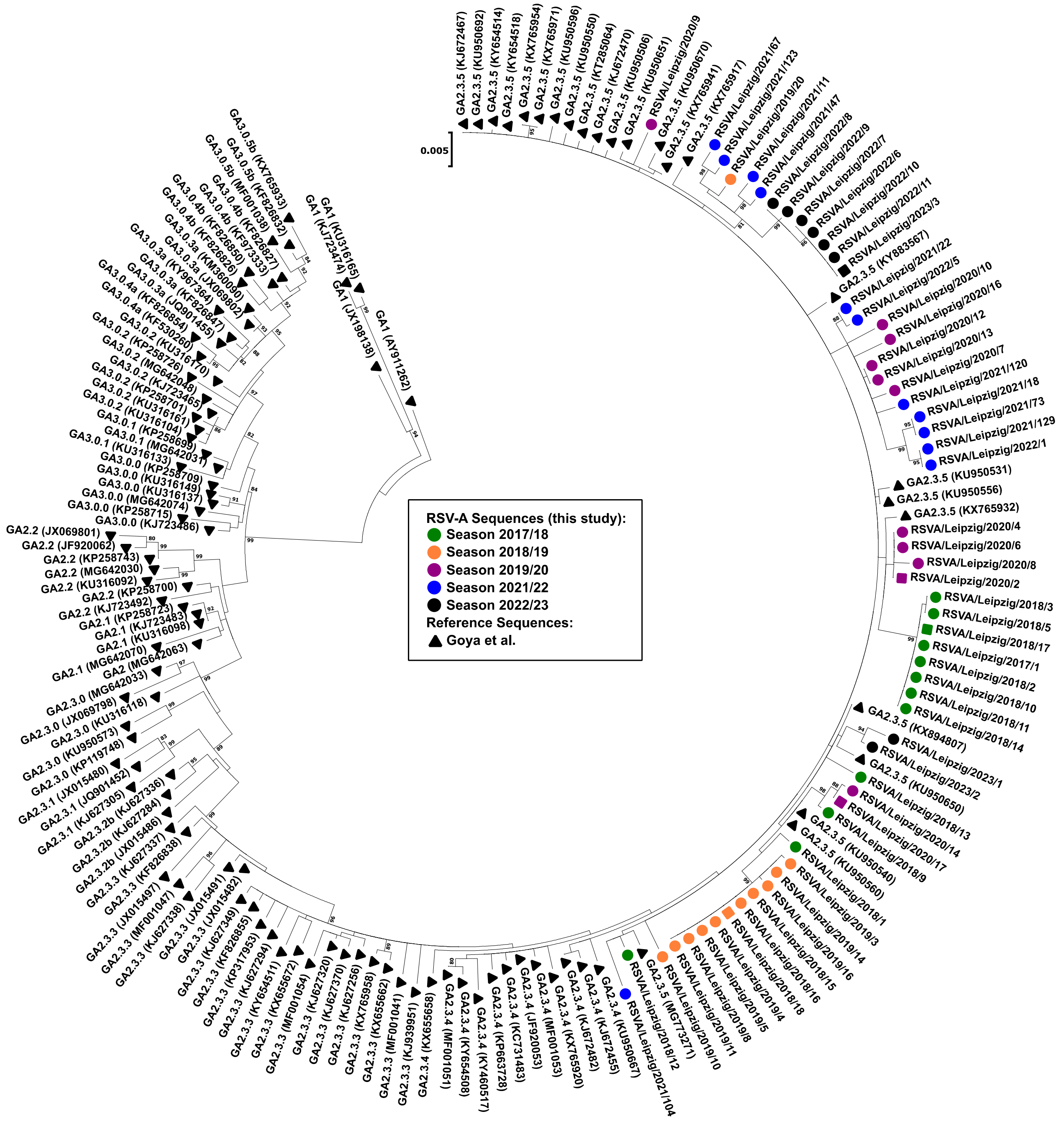


**Figure S1.** Molecular Phylogenetic analysis of the RSV-A F gene by Maximum Likelihood method. The evolutionary history was inferred by using the Maximum Likelihood method based on the Tamura-Nei model [39]. The tree with the highest log likelihood (-8,897.71) is shown. The percentage of trees in which the associated taxa clustered together is shown next to the branches. Initial tree(s) for the heuristic search were obtained automatically by applying Neighbor-Joining and BioNJ algorithms to a matrix of pairwise distances estimated using the Maximum Composite Likelihood (MCL) approach, and then selecting the topology with superior log likelihood value. The tree is drawn to scale, with branch lengths measured in the number of substitutions per site. The analysis involved 171 nucleotide sequences. There was a total of 1,725 positions in the final dataset. Evolutionary analyses were conducted in MEGA7 [38]. Only nodes with a statistical support >80% are shown. The symbols indicate the sequence origin or the season of the indicated strain: dots/squares: green: season 2017/2018 isolates; orange, season 2018/2019 isolates; purple, season 2019/2020 isolates; blue, season 2021/2022 isolates; black, season 2022/2023 isolates; squares, fatal cases; black triangle: consensus reference sequences according to Goya et al. [16].

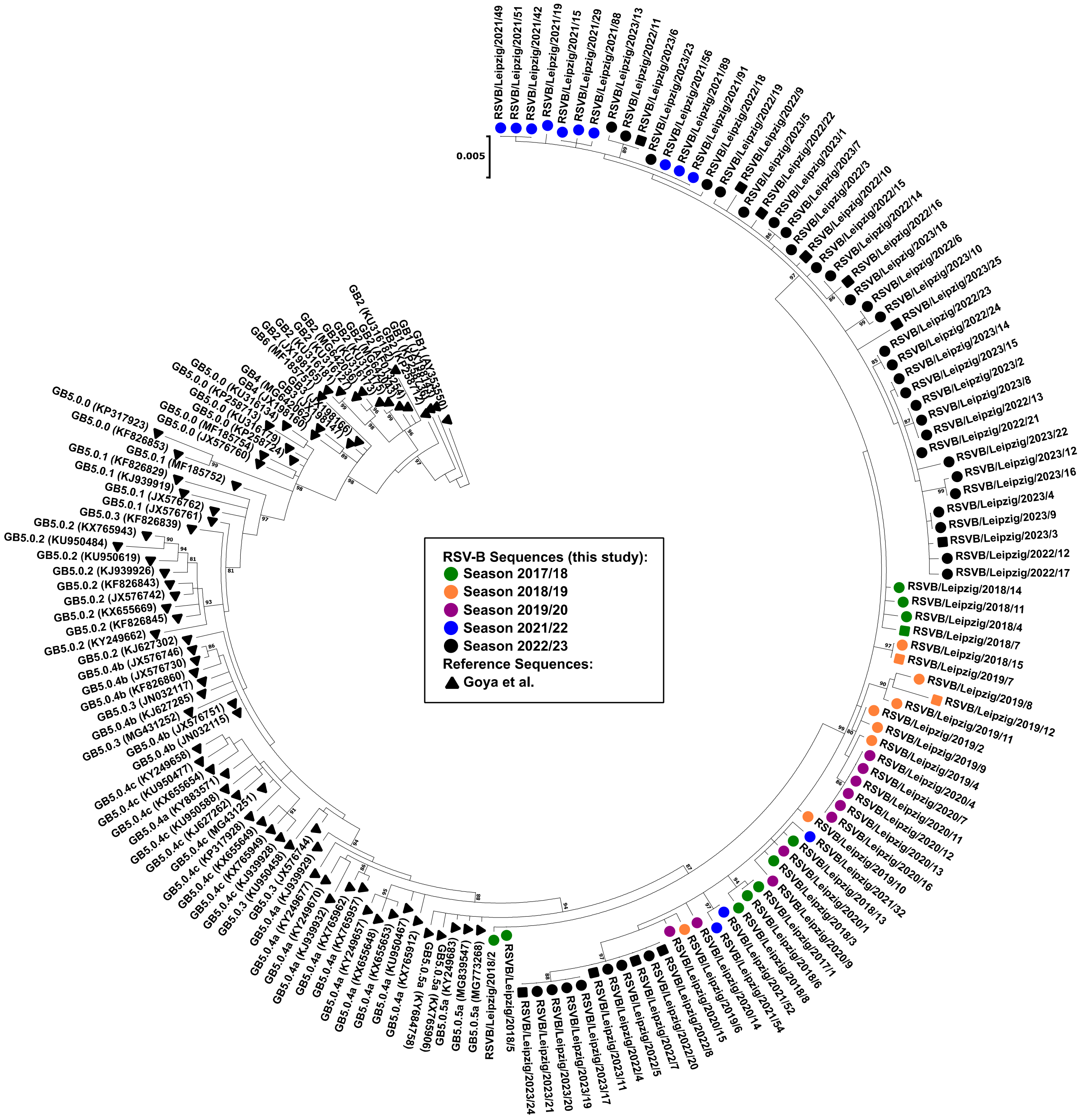


**Figure S2.** Molecular Phylogenetic analysis of the RSV-B F gene by Maximum Likelihood method. The evolutionary history was inferred by using the Maximum Likelihood method based on the Tamura-Nei model [39]. The tree with the highest log likelihood (-7,008.58) is shown. Initial tree(s) for the heuristic search were obtained automatically by applying Neighbor-Joining and BioNJ algorithms to a matrix of pairwise distances estimated using the Maximum Composite Likelihood (MCL) approach, and then selecting the topology with superior log likelihood value. The tree is drawn to scale, with branch lengths measured in the number of substitutions per site. The analysis involved 170 nucleotide sequences. There were a total of 1725 positions in the final dataset. Evolutionary analyses were conducted in MEGA7 [38]. Only nodes with a statistical support >80% are shown. The symbols indicate the sequence origin or the season of the indicated strain: dots/squares: green: season 2017/2018 isolates; orange, season 2018/2019 isolates; purple, season 2019/2020 isolates; blue, season 2021/2022 isolates; black, season 2022/2023 isolates; squares, fatal cases; black triangle: consensus reference sequences according to Goya et al. [16].
